# Molecular Sieve Promoted Growth of Ferroelectric Trilayer 3R‐MoS_2_ for Polarization‐Dependent Reconfigurable Optoelectronic Synapses

**DOI:** 10.1002/advs.202524333

**Published:** 2026-01-20

**Authors:** Qichao Xue, Jincheng Zhang, Yuying Wang, Yu Wang, Kuiwei Li, Yuxuan Chen, Fang Zhong, Qing Li, Ning Zhou, Chenying Yang, Yuchuan Shao, Tao Liang

**Affiliations:** ^1^ Hangzhou Institute for Advanced Study University of Chinese Academy of Sciences Hangzhou China; ^2^ Key Laboratory of Materials for High‐Power Laser Shanghai Institute of Optics and Fine Mechanics Chinese Academy of Sciences Shanghai China

**Keywords:** 2D sliding ferroelectricity, ferroelectric semiconductors, polarization switching, reconfigurable optoelectronic synapse, trilayer rhombohedral‐stack MoS_2_

## Abstract

2D sliding ferroelectric semiconductors are a unique combination of switchable electric polarization and gate/light modulated charge transport, rendering them an appealing platform for multifunctional electronic and optoelectronic devices. However, the currently available 2D sliding ferroelectric semiconductors are limited, and the coupled gate‐light‐polarization interactions in such systems remain to be further explored. Herein, trilayer (3L) rhombohedral‐stacked (3R) MoS_2_ nanoflakes are synthesized via a molecular sieve‐assisted chemical vapor deposition strategy, in which the combined physical vapor buffer and chemical sodium release from the molecular sieves effectively promote vertical growth with a favorable 3R stacking configuration. The noncentrosymmetric crystal structure, switchable out‐of‐plane polarization, and robust sliding ferroelectricity are systematically characterized. Integrating 3L 3R‐MoS_2_ into ferroelectric semiconductor field‐effect transistors (FeS‐FETs) reveals a large polarization‐direction‐dependent memory window of 13.8–14.6 V. Moreover, the ferroelectric polarization state, reversibly controlled by the polarity of the gate voltage, strongly governs the temporal photoresponse, enabling reconfigurable progressive photocurrent depression or facilitation. Combined with its narrow bandgap and defect‐assisted absorption, a broadband optoelectronic synaptic plasticity was further evaluated under a specific polarization state. These results establish 3L 3R‐MoS_2_ as a highly promising 2D crystals for ferroelectric optoelectronic synapses and future in‐memory sensing systems.

## Graphical abstract

The noncentrosymmetric trilayer rhombohedral‐stacked MoS_2_ nanoflakes with enhanced sliding ferroelectric properties are synthesized via a molecular sieve‐assisted chemical vapor deposition process. The switchable polarization states, combined with the exceptional light/gate voltage modulated electrical properties of these nanoflakes, enable broadband and reconfigurable neuromorphic optoelectronic synaptic devices.

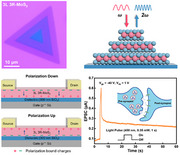

## 1 Introduction

Ferroelectric semiconductors are an emerging class of materials that exhibit switchable spontaneous polarization and semiconducting properties simultaneously. The coexistence of electric dipoles and mobile charge carriers enables their integration into novel device architectures, such as ferroelectric semiconductor field‐effect transistors (FeS‐FETs) [1], non‐volatile memory devices [2], and neuromorphic computing systems [3]. Unlike conventional ferroelectric FETs (Fe‐FETs), which rely on a ferroelectric insulating layer to modulate the conductance of a semiconducting channel, FeS‐FETs utilize a ferroelectric semiconductor directly as the channel material. In such devices, the carrier transport is intrinsically coupled to the polarization state of the channel, enabling effective screening of depolarization fields and suppressing leakage currents that typically compromise Fe‐FETs performance [1, 4, 5]. Additionally, the electrical properties of ferroelectric semiconductors are highly sensitive to intrinsic structural defects and external stimuli, which provide a versatile platform for investigating the complex interaction between electric/light‐driven free carriers and bound polarization charges. As advanced integrated circuits are continuously miniaturized, conventional ferroelectric oxides face critical challenges, including difficulties in downscaling thickness, interface instability, and integration complexity. In this context, 2D van der Waals (vdW) ferroelectric materials, featuring atomic thickness and dangling‐bond‐free surfaces, have emerged as promising alternatives for next‐generation complementary metal‐oxide‐semiconductor (CMOS) integration [6, 7, 8].

2D vdW ferroelectrics share the same fundamental polarization mechanisms as conventional bulk ferroelectrics and are generally classified as displacive (e.g., α‐In_2_Se_3_) [9, 10] or order‐disorder (e.g., CuInP_2_S_6_) [11, 12] type, or a combination of them. However, they exhibit several distinctive features that are absent in conventional systems. For instance, due to the mobile Cu atoms, 2D CuInP_2_S_6_ exhibits a quadruple‐well Gibbs free‐energy landscape [13], in contrast to the classical double‐well potential described by Landau–Ginzburg theory. In another 2D ferroelectric semiconductor α‐In_2_Se_3_, a dipole‐locking effect is observed [10, 14], in which the out‐of‐plane and in‐plane polarization states switch simultaneously via a coupled mechanism, even when thinned to the monolayer limit [15]. Moreover, as α‐In_2_Se_3_ possesses a direct bandgap of ∼1.3 eV [8, 16], which is suitable for photodetection, its photocurrent can be modulated by ferroelectric polarization [17], and conversely, photogenerated carriers can influence the distribution of screening charges and even reverse the polarization direction [18, 19]. Recently, interlayer sliding ferroelectricity has emerged as a fundamentally distinct polarization mechanism in 2D vdW materials such as BN [20, 21], InSe [22, 23], and transition metal dichalcogenides (TMDs) [24, 25, 26, 27, 28]. In these systems, spontaneous polarization arises from relative in‐plane displacements between adjacent layers that can break inversion symmetry. This mechanism enables ultrafast and fatigue‐free switching without the need for inherently polar crystal structures, and features a low energy barrier for polarization reversal. Among them, bilayer (2L) TMDs serve as prototypical platforms to explore sliding ferroelectricity [25, 26, 27, 28, 29, 30, 31]. In the hexagonal‐stacked (2H or AB‐stacked) configuration, inversion symmetry is preserved due to the vertical alignment of metal and chalcogen atoms from adjacent layers, and thereby the ferroelectricity is largely suppressed. In contrast, rhombohedral‐stacked (3R or AA‐stacked) bilayers break inversion symmetry via in‐plane offsets, leading to aligned in‐plane dipoles, enhanced second harmonic generation (SHG), and robust out‐of‐plane ferroelectric polarization.

Despite the demonstrated ferroelectricity in 3R‐stacked bilayers, their integration with optoelectronic functionality remains to be further explored. Furthermore, extending the 3R stacking sequence to trilayer (3L) TMDs introduces an additional degree of freedom via interlayer translation and twist, while maintaining a noncentrosymmetric crystal structure [32]. Compared to their monolayer (1L) and 2L counterparts, 3L 3R‐TMDs exhibit narrower bandgaps for broadband optical absorption [33], enhanced electrical conductivity [34], and reduced contact resistance with metal electrodes [35]. These attributes, together with electrically tunable polarization states, make 3L 3R‐TMDs compelling candidates for reconfigurable ferroelectric devices, such as neuromorphic optoelectronic synapse capable of emulating biological vision and signal processing under both electrical and optical stimuli [36, 37]. Nevertheless, their intrinsic ferroelectric switching behavior and full potential in multifunctional optoelectronic applications have yet to be systematically explored.

In this work, we report the synthesis of 3L 3R‐stacked MoS_2_ nanoflakes via a molecular sieve promoted CVD process. Leveraging the fact that elevated local molybdenum concentrations promote multilayer nucleation and growth, the introduction of molecular sieves facilitates the accumulation of molybdenum species near the substrate surface, thereby enhancing the out‐of‐plane growth of 3L 3R‐stacked MoS_2_. The high phase purity, noncentrosymmetric structure, and ferroelectric properties are thoroughly evaluated with spectroscopic and electrical transport tests. The reconfigurable optoelectronic synaptic devices depending on the polarization states of this kind of 2D sliding ferroelectric semiconductor are also realized. Our findings highlight the rich functionalities of 3L 3R‐MoS_2_ and position it as a potential platform for developing reconfigurable, light‐sensitive neuromorphic devices and multifunctional memory systems.

## 2 Results and Discussion

### 2.1 Molecular Sieve Promoted Growth of 3R‐MoS_2_ Nanoflakes

**FIGURE 1:**
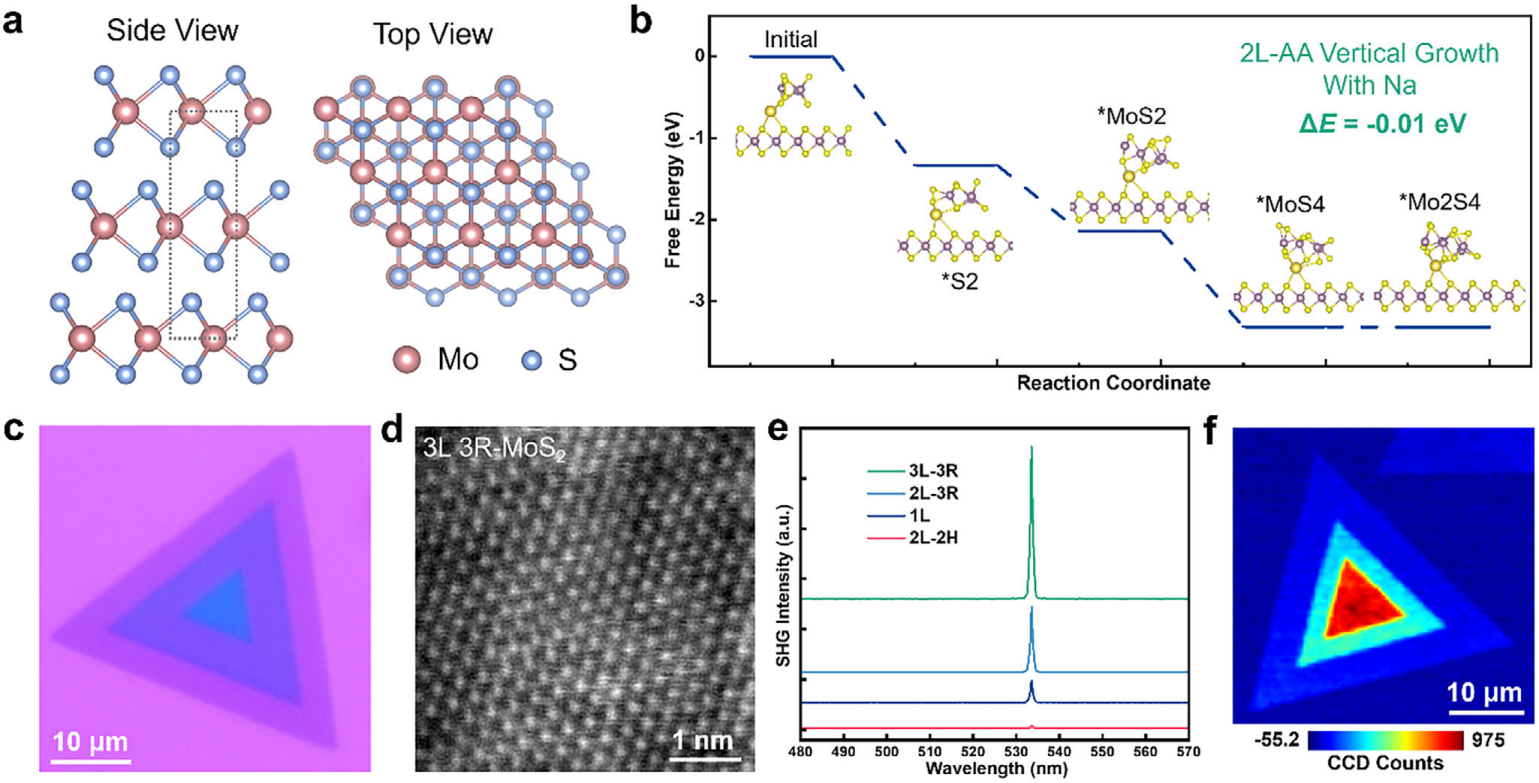
Molecular sieve‐promoted CVD growth and characterization of 3L 3R‐stacked MoS_2_. (a) Side and top views of the atomic structure of the 3L 3R‐stacked MoS_2_. (b) DFT calculation of the growth process of 2L 3R‐stacked MoS_2_ with Na adsorption, showing an energy favorable growth process (ΔE =−0.01 eV). (c) OM image of the CVD‐grown 3L 3R‐stacked MoS_2_. (d) STEM image of the 3L 3R‐MoS_2_ nanoflake. (e) The SHG spectra acquired from the 3R‐stacked 3L, 2L, and 1L regions, as well as that from the 2H‐stacked 2L MoS_2_ nanoflake for comparison. (f) SHG intensity mapping of one 3R‐stacked 3L MoS_2_ nanoflake.

The atomic structure of 3R‐MoS_2_ is illustrated in both side and top views in Figure 1a. In this stacking configuration, three MoS_2_ monolayers are arranged with identical crystallographic orientation but laterally shifted by one‐third of the unit cell along the armchair direction between adjacent layers. This interlayer sliding breaks both inversion symmetry and out‐of‐plane mirror symmetry, which is a structural prerequisite for realizing nonlinear optics and ferroelectricity. The CVD growth of 3R‐MoS_2_ was achieved via a molecular sieve‐promoted process (see Experimental Section). Specifically, the growth substrate was placed face‐down above the precursor to form a confined space that promotes localized molybdenum vapor accumulation (Figure S1). Without the introduction of molecular sieves, only monolayer MoS_2_ nanoflakes were observed, as shown in Figure S2a,c. In contrast, upon evenly spreading molecular sieve powders atop the MoO_3_, additional MoS_2_ layers emerged preferentially at the centers of the triangular monolayer domains (Figure S2b,d), suggesting a transition to vertical growth. Similar phenomena were also observed during the growth of other TMDs including WSe_2_, WS_2_, and MoSe_2_ on diverse substrates such as Si_3_N_4_, sapphire (Al_2_O_3_), and SiO_2_/Si (Figures S3 and S4), demonstrating the universality of the molecular sieve‐promoted strategy for achieving predominantly 3R‐stacked TMD layers.

The molecular sieves, composed of crystalline aluminosilicate (K_n_Na_12‐n_[(AlO_2_)_12_(SiO_2_)_12_]), consist of micrometer‐sized cubic particles with uniform nanopores (Figure S5), which adsorb and gradually release volatile Mo precursors, stabilizing the local supersaturation near the substrate and promoting multilayer growth. Control experiments using porous SiO_2_ or Al_2_O_3_ powders yielded no multilayer 3R‐MoS_2_ (Figure S6), confirming the critical role of alkali metal released from the sieves. Energy dispersive spectrometer (EDS) and X‐ray photoelectron spectroscopy (XPS) characterizations verified that the relative Na content in molecular sieves was reduced after growth (from 7.7 to 3.7 at%, Figure S7), and a prominent Na 1s XPS signal appeared on the substrate surface (Figure S8). Density functional theory (DFT) calculations revealed that Na adatoms lower the energy barrier for monolayer MoS_2_ growth from 0.34 to 0.30 eV (Figure S9), and substantially reduce the energy barriers for growth of 2L 2H (from 0.13 to 0.04 eV) and 2L 3R (from 0.81 to −0.01 eV) stacking (Figure 1b; Figure S10), making the 3R configuration thermodynamically favored. Therefore, molecular sieves act as both a physical vapor buffer and a chemical sodium source [38, 39], jointly enabling dominant 3R stacking.

The 3R‐stacking configuration of the grown nanoflakes, especially the 3L nanoflakes, was confirmed through a complementary set of techniques. Optical microscopy (OM) image of a typical 3L 3R‐MoS_2_ nanoflake (Figure 1c) shows a lateral size of ∼ 40 µm with a distinct stepped morphology, consistent with the atomic force microscope (AFM) height profile (Figure S11). Raman and photoluminescence (PL) spectra further verify the layer‐dependent properties, showing an increased separation between the characteristic Raman modes and a transition from a direct to an indirect bandgap as the thickness increases from 1L to 3L (Figure S12). Scanning transmission electron microscope (STEM) imaging of the 3L region (Figure 1d) reveals a well‐ordered hexagonal lattice consistent with the structural model in Figure 1a. Comparative STEM and selected area electron diffraction (SAED) analyses of the 1‐3L regions (Figure S13) further confirm the presence of the 3R‐stacking configuration in both the 2L and 3L regions of the 3L 3R‐MoS_2_ nanoflake.

### 2.2 Broken Inversion Symmetry and Ferroelectricity

The optical second harmonic generation (SHG) is a nonlinear frequency doubling process sensitive to symmetry breaking and serves as a powerful probe for identifying noncentrosymmetric crystal structures. Given the absence of inversion symmetry in 3R‐stacked MoS_2_ nanoflakes, SHG spectroscopy was employed to confirm the structural and optical characteristics of the synthesized nanoflakes (Figure S14a). A 1064 nm femtosecond laser with a 6 ps pulse width was used as the excitation source. Strong SHG signals were observed at 532 nm from the 3R‐stacked 3L, 2L, and 1L regions (Figure 1e), corresponding to the frequency doubling of the excitation wavelength. The SHG intensity for an *N*‐layer 3R‐MoS_2_ crystal can be described by the following Equation (1) [40]:

(1) $$I\left(2\omega \right)={\left|E\left(2\omega \right)\right|}^{2}=C^{2}N^{2}\chi_{MoS_{2}}^{\left(2\right)}{\left(\omega \right)}^{2}cos^{2}\left(3\Phi +\Phi_{0}\right)$$

where *C* is a proportionality constant accounting for local field factors, *N* is the number of layers, *Φ* is the polarization angle of the incident light, and *Φ*_0_ is the initial crystallographic orientation. The observed quadratic dependence of SHG intensity on *N* aligns well with Equation (1) and is further supported by fitting results in Figure S14b. This quadratic scaling confirms the uniform 3R stacking sequence across the 3L and 2L regions. To highlight the role of stacking symmetry, SHG measurements were also performed on 2H‐stacked 2L MoS_2_ samples with antiparallel triangular orientation under identical conditions (Figure S14c). The SHG response from the 2H‐stacked region was significantly suppressed (Figure 1e), consistent with its recovered centrosymmetric nature and confirming the absence of net polarization. In the 3L 3R‐MoS_2_ region, the SHG intensity shows a clear power‐dependent increase that follows a parabolic‐like trend (Figure S14d,e), as expected for a second‐order nonlinear optical process. Moreover, the polarization‐resolved SHG pattern exhibits a sixfold rotational symmetry (Figure S14f), in agreement with the threefold crystal symmetry. SHG mapping of the nanoflake (Figure 1f) reveals a monotonic increase in SHG intensity from the 1L edge toward the 3L center, consistent with the spectral results. The spatially uniform SHG response within each layer further supports the high crystalline quality, phase purity, and broken inversion symmetry of the synthesized MoS_2_ nanoflakes.

**FIGURE 2:**
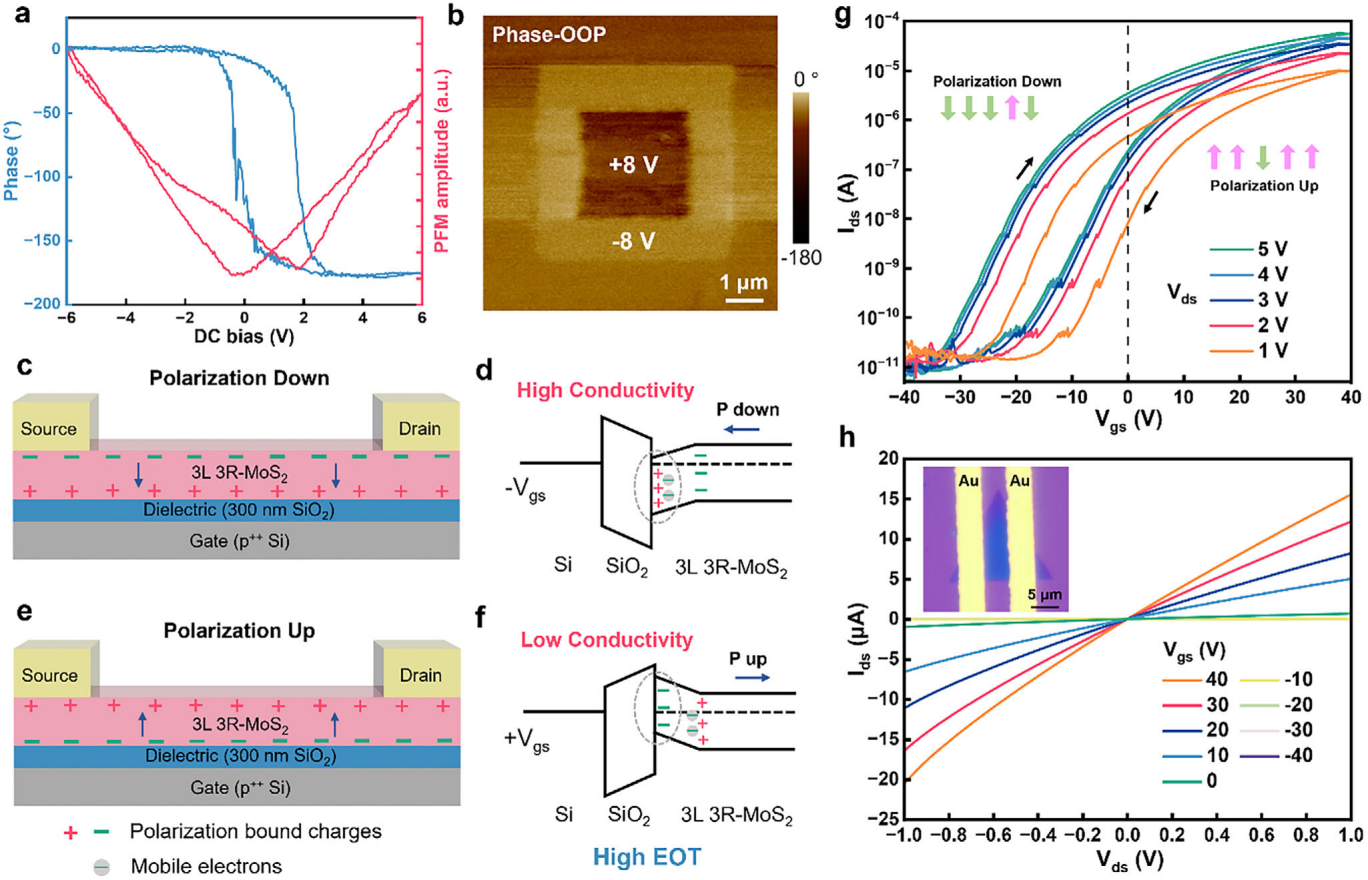
PFM tests and FeS‐FETs based on 3L 3R‐MoS_2_ nanoflakes. (a) The local PFM phase and amplitude loops during the switching process. (b) The out‐of‐plane (OOP) PFM phase after writing with forward (+8 V) and reverse (−8 V) DC bias in the box pattern. (c, d) The polarization down state is achieved when a negative *V*_gs_ lower than the coercive voltage is applied. The channel bottom distributes positive polarized bound charges, the energy band is bent downward, and electrons are accumulated to induce a high conductivity. (e, f) The polarization up state is achieved when a positive *V*_gs_ higher than the coercive voltage is applied. The channel bottom distributes negative polarized bound charges, the energy band is bent upward, and the electrons are depleted to form a low conductivity. (g) Transfer characteristic curves of the 3L 3R‐MoS_2_ FeS‐FETs showing large clockwise hysteresis windows at *V*_ds_ ranging from 1 to 5 V. The vertical arrows represent the gradually switched polarization direction during the *V*_gs_ sweeping process. On the left side (negative *V*_gs_), the downward polarization direction dominates while the upward polarization dominates on the right side (positive *V*_gs_). (h) The output characteristic curves of the FeS‐FETs devices. Inset shows the OM image of the device for transfer and output curves test.

To validate the presence of switchable out‐of‐plane polarization in 3L 3R‐MoS_2_, the as‐grown nanoflakes were transferred onto a conductive Au/SiO_2_ substrate for piezoresponse force microscopy (PFM) measurements. As shown in Figure 2a, the PFM amplitude and phase hysteresis loops display pronounced butterfly and 180 ° phase‐switching behavior under a varying electric field, confirming the existence of switchable ferroelectric polarization. The hysteresis loops from multiple 3L 3R‐MoS_2_ nanoflakes show consistent profiles with a coercive voltage of ∼ 1 V (Figure S15), confirming the reproducibility of their ferroelectric response. In contrast, 2L 3R‐MoS_2_ exhibits weaker yet observable polarization switching (Figure S16a), while 2L 2H‐MoS_2_ and 1L MoS_2_ show no ferroelectricity due to their centrosymmetric or mirror‐symmetric structures (Figure S16b,c). These comparisons highlight that the 3R stacking order is essential for breaking inversion symmetry and inducing spontaneous polarization. Distinct amplitude and phase contrasts were observed in regions written with +8 and −8 V bias, indicating clear and reversible polarization states (Figure 2b; Figure S17a). The contrasts remain discernible even after 60 min in air (Figure S17c–h). The gradually blurred contrast was attributed to the polarization screened by the thermally‐activated free carriers at room temperature in this kind of semiconductors, as well as the carrier injection/leakage from the Au substrate. Furthermore, a vertical graphene/3L 3R‐MoS_2_/graphene device exhibits a clear hysteretic *I*_ds_‐*V*_ds_ loop (Figure S18), further validating the polarization switching behavior. These findings unambiguously establish the 3L 3R‐MoS_2_ as a robust ferroelectric semiconductor. The coexistence of spontaneous polarization and mobile charge carriers enables its direct use as an active channel material in FeS‐FETs, where both the polarization state and carrier density can be simultaneously modulated by an external gate voltage (*V*_gs_) and light illumination.

### 2.3 Ferroelectric Semiconductor Field‐Effect Transistors (FeS‐FETs)

To investigate the ferroelectric semiconducting behavior in device configurations, FeS‐FETs were constructed by transferring the CVD‐grown 3L 3R‐MoS_2_ nanoflakes onto a SiO_2_(300nm)/Si substrate. The out‐of‐plane polarization direction of the MoS_2_ channel can be switched by the applied positive or negative gate voltage. The heavily doped silicon substrate functions as a global back gate, while the 300 nm SiO_2_ layer serves as the gate dielectric. The relatively thick dielectric corresponds to a high equivalent oxide thickness (EOT), which generates a weak vertical electric field. Consequently, the gate field predominantly modulates the carrier concentration near the bottom surface (BS) of the MoS_2_ channel. To minimize Fermi level pinning and avoid damage from conventional high‐energy metal deposition, Au source and drain electrodes were mechanically transferred onto the MoS_2_ channel [41]. When a negative *V*_gs_ is applied, positive bound polarization charges accumulate at the BS, stabilizing the polarization‐down state (Figure 2c). This additional electrostatic field attracts electrons toward the BS, inducing downward band bending (Figure 2d) and rendering the device high conductivity. Conversely, a positive *V*_gs_ results in negative bound charges at the BS, establishing a polarization‐up state. This configuration repels electrons from the BS and leads to upward band bending and low conductivity (Figure 2e,f). Due to the weak penetration of the gate field through the multilayer channel, the top surface of the MoS_2_ channel does not contribute significantly to the conduction (Figure S19) [1]. The transfer characteristics of the 3L 3R‐MoS_2_ FeS‐FETs under bidirectional *V*_gs_ sweeps at individual source‐drain voltages (*V*_ds_) are presented in Figure 2g. All curves exhibit prominent clockwise hysteresis loops, which are attributed to the combined effects of partial polarization switching confined near the BS and charge trapping/detrapping at the MoS_2_ surface (Figure S20). A notable memory window extracted at a source‐drain current (*I*_ds_) of 10 nA exhibits a narrow distribution of 13.8–14.6 V for *V*_ds_ ranging from 1 to 5 V, significantly exceeding the performance benchmarks of previously reported FeS‐FETs based on 2D α‐In_2_Se_3_ [42] and 3R‐stacked 2L MoS_2_ [27, 28]. The output curves (Figure 2h) confirm a Schottky contact between MoS_2_ and the transferred Au electrodes. Similar electrical behavior was observed in devices using a uniform 3L 3R‐MoS_2_ channel (Figure S21), confirming that the ferroelectric polarization switching is an intrinsic property of the 3R‐stacked 3L MoS_2_, independent of channel morphology or contact configuration. The FeS‐FET devices demonstrate an on/off current ratio of ∼ 7.9 × 10^6^, electron mobility of ∼ 7.5 cm^2^ V^−1^ s^−1^ at *V*_ds_ = 1 V, and an on‐state current of 3.86 µA µm^−1^ at *V*_ds_ = 5 V. These electrical metrics of 3L 3R‐MoS_2_ are attributed to its reduced bandgap, increased carrier concentration, and lowered contact resistance.

### 2.4 Polarization‐Dependent Reconfigurable Optoelectronic Synapses

**FIGURE 3:**
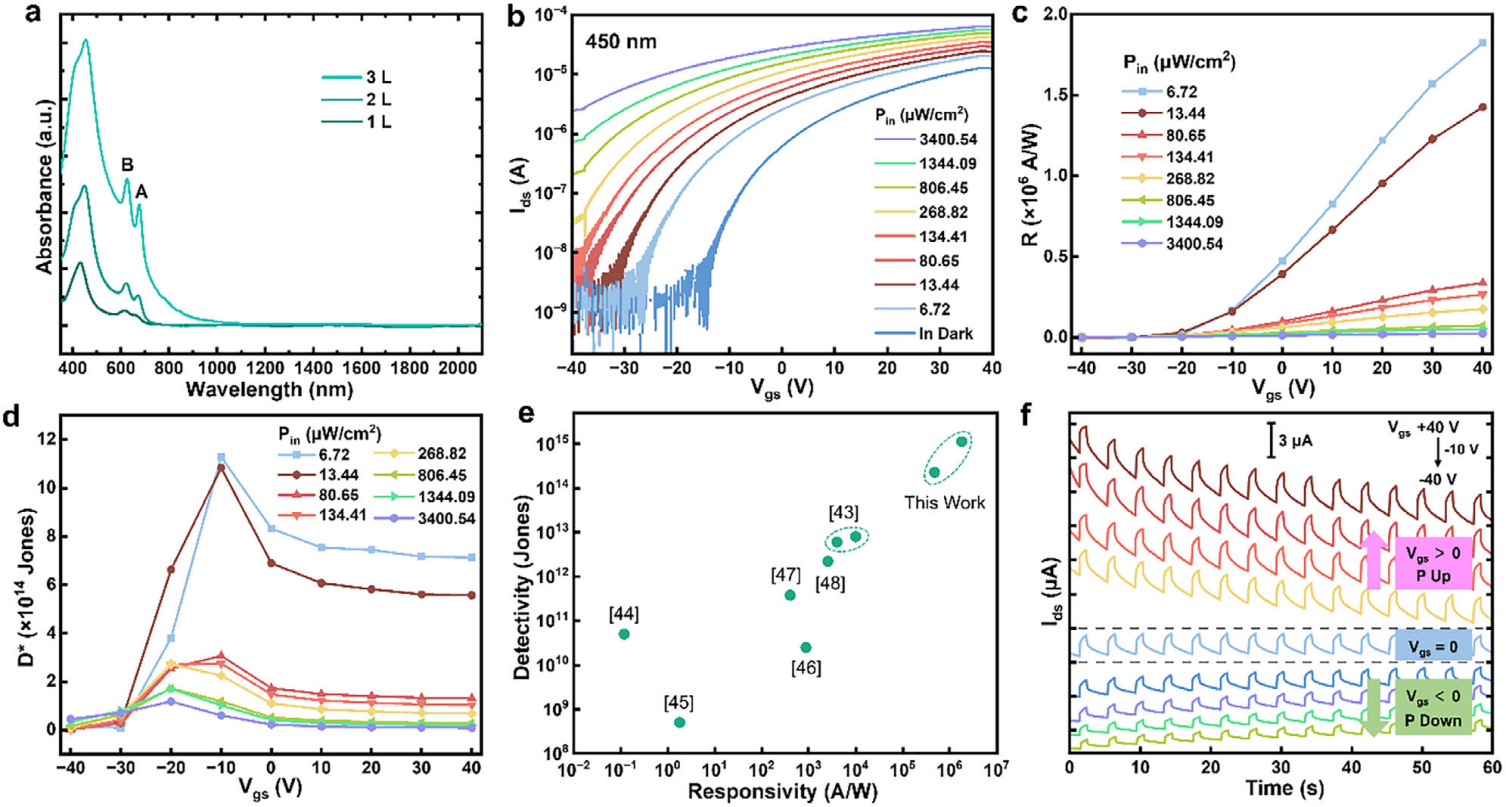
Reconfigurable PPF/PPD modulated by the *V*_gs_ (polarization direction). (a) Micro‐zone absorbance curves acquired from the 1L, 2L, and 3L regions of the same 3L 3R‐MoS_2_ nanoflake. (b) Transfer characteristics of the FeS‐FET under 450 nm illumination with increasing incident power densities. *V*_ds_ = 1 V. (c, d) The calculated (c) *R* and (d) D^*^ from (b) as a function of *V*_gs_. (e) Comparison of *R* and D^*^ for photodetectors based on 3L 3R‐MoS_2_ in this work and other reported CVD synthesized MoS_2_ layers. (f) Temporal response of the device under varied *V*_gs_ from −40 to 40 V. A declining trend of *I*_ds_ is observed for positive *V*_gs_ (polarization up) while a rising trend in *I*_ds_ is observed for negative *V*_gs_ (polarization down).

In the 3L 3R‐MoS_2_ FeS‐FETs, the channel carrier concentration and polarization directions are simultaneously modulated by the *V*_gs_. Specifically, sweeping *V*_gs_ not only tunes the electron density in the n‐type MoS_2_ channel, where a more positive *V*_gs_ induces higher electron accumulation, but also switches the out‐of‐plane polarization between upward and downward states under positive and negative *V*_gs_, respectively. As a result, the light‐matter interaction in these devices becomes intrinsically polarization dependent. Furthermore, the micro‐zone absorption spectra of 1–3 L regions in 3R‐MoS_2_ (Figure 3a) show increasing absorption intensity and a redshift of the absorption edge from ∼ 700 nm (1L) to ∼ 1100 nm (3L), indicating bandgap narrowing with thickness, which is anticipated to exhibit broadband photodetection performance. To assess these behaviors, transfer and output curves were measured in dark and under laser illumination of varying wavelengths and power densities. Taking the 450 nm laser as an example, a pronounced negative shift in threshold voltage (*V*_th_) is observed, from approximately ‐15 V in dark to ‐40 V under increasing illumination intensity (Figure 3b). This shift evidences a strong n‐type photogating effect whereby a more negative *V*_gs_ is required to fully deplete both intrinsic and photoinduced carriers. Importantly, a substantial photocurrent is generated over a wide *V*_gs_ range from −40 to +40 V even under a weak illumination intensity (6.72 µW/cm^2^). The photoresponsivity (*R*) is plotted as a function of *V*_gs_ in Figure 3c, in which *R* increases monotonically with increasing *V*_gs_, reflecting the exponential rise in *I*_ds_ relative to *I*_dark_ under stronger gate‐induced accumulation. A maximum *R* of 1.8 ×10^6^ A/W is obtained at *V*_gs_ = +40 V and *P*_in_ = 6.72 µW/cm^2^. The external quantum efficiency (*EQE*), calculated as *EQE* = *Rhc*/*eλ*, is directly proportional to *R* and peaks at 5.04×10^8^ % under the same conditions (Figure S22), highlighting the presence of strong photoconductive gain mechanisms. The specific detectivity (*D* ^*^) was further evaluated using *D* ^*^ = *RS* ^1/2^/(2*eI*_dark_)^1/2^ assuming that shot noise from dark current is the major contributor to the total noise, where *S* is the active area of illumination. As shown in Figure 3d, *D* ^*^ exhibits a non‐monotonic dependence on *V*_gs_ with a peak value of 1.13 × 10^15^ Jones at *V*_gs_ = ‐10 V under 6.72 µW/cm^2^ excitation. The devices also exhibit a high photodetection performance in the depleted *V*_gs_ region. For example, at *V*_gs_ = −20 V under 6.72 µW/cm^2^ excitation, the *R*, *EQE*, and *D* ^*^ are 1.77 × 10^4^ A/W, 4.89 × 10^6^ %, and 3.81 × 10^14^ Jones, respectively, guaranteeing a highly sensitive photodetector with a low dark current. Note that in the negative to positive *V*_gs_ sweep, the MoS_2_ channel experiences a polarization direction switching from downward to upward. The photodetection metrics as a function of incident light power density are also shown in Figure S22. Under visible excitation with longer wavelengths (637 and 785 nm), the photodetector performance degrades slightly (Figures S23 and S24). For example, under 637 nm illumination, *R*, *EQE*, and *D* ^*^ values of 4.78 × 10^5^ A/W, 9.31 × 10^7^ %, and 2.28 × 10^14^ Jones are obtained, respectively. These values exceed those reported for previously studied 2D MoS_2_‐based phototransistors (Figure 3e; Table S1) [43, 44, 45, 46, 47, 48]. As the excitation wavelength extends further, especially into the near‐infrared (NIR) region (1060 and 1310 nm), a measurable photoresponse still persists despite the device metrics decline (Figures S25, S26, and S27). DFT calculations confirm an indirect bandgap of ∼ 1.1 eV for 3L 3R‐MoS_2_ (Figure S28), consistent with the absorbance curves. The weak photocurrent observed at 1310 nm is attributed to sulfur‐vacancy‐induced mid‐gap states (∼ 0.2 eV below the conduction band minimum) revealed by STEM images and DFT (Figures S29 and S30), which enable sub‐bandgap absorption. Such defect‐assisted NIR photoresponse was also observed in 1L MoS_2_, 2L 3R‐MoS_2_, and 3L 2H‐MoS_2_ devices (Figures S31–S33).

The temporal photoresponse of the device under different *V*_gs_ is presented in Figure 3f, where distinctly different evolution trends of the *I*_ds_ are observed. Under a constant positive *V*_gs_, corresponding to an upward polarization state of the MoS_2_ channel, *I*_ds_ exhibits a gradual decay with increasing light pulse number. In contrast, when a constant negative *V*_gs_ is applied, the device shows an opposite behavior, with *I*_ds_ progressively increasing upon successive light pulses. This gate‐tunable transition between progressive photocurrent depression (PPD)/facilitation (PPF) originates from the ferroelectric polarization of the 3R‐stacked MoS_2_ channel, i.e., the polarization direction modulates the electron concentration and the channel conductivity, leading to high‐ and low‐conductivity under opposite *V*_gs_. The transition can be further understood from the band structures diagrams (Figure S34). Intrinsic defects, such as molybdenum and sulfur vacancies, introduce mid‐gap states that act as localized recombination centers and trap states, which profoundly influence the device's photoresponse [47]. Recombination centers, typically situated near the mid‐gap, facilitate rapid electron‐hole recombination, thereby reducing photoconductive gain and responsivity. In contrast, trap states positioned near the conduction or valence band edges can transiently capture charge carriers, with their trapping durations governed by the trap depth and energy [47]. Under negative *V*_gs_ (polarization down), the Fermi level shifts away from the conduction band, leaving a large population of unoccupied trap states above it (Figure S34a). Upon light illumination, photogenerated carriers are partially captured by these traps (Figure S34b). When the illumination ceases, the slow release of trapped carriers results in PPF. In contrast, under positive *V*_gs_ (polarization up), the Fermi level moves closer to the conduction band, leading to the occupation of most available trap states (Figure S34c). Under weak illumination, deep and long‐lived hole traps dominate the photocurrent dynamics, enabling high photogain and *R*. However, as the illumination intensity increases, shallower traps with shorter lifetimes become increasing populated (Figure S34d). These shallow traps promote faster carrier recombination, thereby decreasing photoconductive gain. The recombination of intrinsic electrons with previously trapped holes leads to the PPD upon repeated light exposure. This reconfigurable PPF/PPD states via *V*_gs_ modulation (polarization direction switching) within a single device underscores the advantage of 3L 3R‐MoS_2_ for multifunctional excitatory and inhibitory synaptic functions. The similar phenomenon was also observed when the 637 nm laser was illuminated onto the device under positive and negative *V*_gs_ (Figure S35).

**FIGURE 4:**
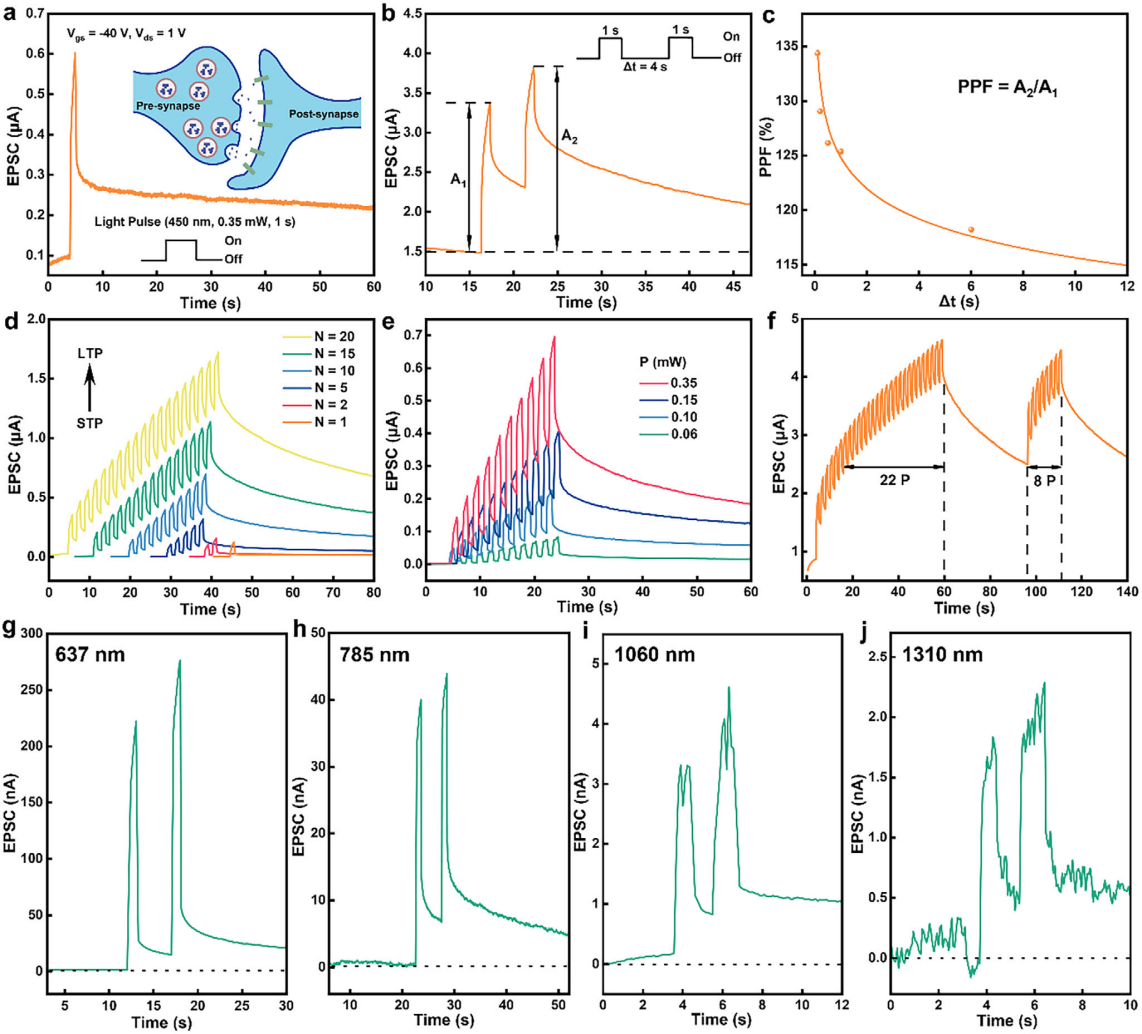
Neuromorphic optoelectronic synaptic performance of 3L 3R‐MoS_2_ device at *V*_gs_ = −40 V. (a) EPSC upon a single light pulse is applied to the device at *V*_ds_ = 1 V. (b) The PPF effect when a pair of light pulses with an interval of 4 s is applied to the device. (c) PPF ratio as a function of the pulse interval. (d, e) The plasticity transition from STP to LTP by increasing (d) the pulse number (*N*) and (e) the laser power. (f) Learning, forgetting, and relearning processes emulated by the device. (g–j) The broadband PPF behaviors when incident laser wavelengths of 637, 785, 1060, and 1310 nm are used.

The artificial optoelectronic synapse performance based on 3L 3R‐MoS_2_ FeS‐FET devices were evaluated in detailed at *V*_gs_ = −40 V (polarization down state), in which a lower dark current and higher PPF metrics are expected. In biological synapse, neurotransmitters released from the presynapse neuron induce an excitatory postsynaptic current (EPSC) in the postsynaptic neuron (inset of Figure 4a). Analogously, in our devices, an optical pulse is used as the presynaptic stimulus, while the *I*_ds_ functions as the EPSC. As shown in Figure 4a, 1 s light pulse induces a rapid increase in *I*_ds_, followed by a gradual decay upon cessation of illumination. Notably, the post‐stimulus current does not return to its initial baseline, indicating the presence of PPF effect. When a pair of light pulses with an interval time (Δt) of 4 s was applied, the EPSC triggered by the second pulse (A_2_) exceeds that of the first (A_1_), indicative of paired‐pulse facilitation (PPF), which arises from residual photogenerated carriers (Figure 4b). The PPF index, defined as PPF = A_2_/A_1_, gradually declines with increasing Δt (Figure 4c), consistent with the memory fading effect observed in biological synapses. This decay behavior can be modeled using a double exponential function equation (2),

(2) $$PPF=1+C_{1}\times exp\left(-\frac{\Delta t}{\tau_{1}}\right)+C_{2}\times exp\left(-\frac{\Delta t}{\tau_{2}}\right)$$

where *C*_1_ and *C*_2_ are the initial facilitation amplitude, and *τ*_1_ and *τ*_2_ correspond to the fast and slow relaxation time constants, respectively. The fitting results yield *τ*_1_ = 0.09 s and *τ*_2_ = 20.4 s, mirroring short‐ and long‐term synaptic dynamics in biological systems, respectively. To emulate long‐term plasticity (LTP), the number (*N*) and intensity of optical pulses were systematically varied. As shown in Figure 4d,e, increasing either parameter leads to a pronounced enhancement of EPSC, demonstrating a transition from STP to LTP, analogous to the learning process in the human brain. The processes of learning, forgetting, and relearning were simulated by light pulses in Figure 4f. During the initial learning, a series of optical pulses gradually elevates the EPSC, representing memory formation. Upon cessation of the stimuli, the current decays, simulating memory fading. When less light pulses are re‐applied to simulate the relearning process, a faster recovery of EPSC is observed, implying a memory retention effect similar to that of biological synapse, where previous learning accelerates relearning. Owing to the broadband photoresponse of the 3L 3R‐MoS_2_ channel, the device functions effectively as an optoelectronic synapse under diverse wavelengths. As shown in Figure 4g,h and Figures S36 and S37, visible light excitation (637 nm and 785 nm) induces strong synaptic responses. Moreover, even under near‐infrared stimuli (1060 and 1310 nm, Figure 4i,j; Figures S38 and S39), a measurable synaptic output persists. Collectively, these results highlight the potential of 3L 3R‐MoS_2_ as broadband optoelectronic synapses reconfigured by *V*_gs_ (polarization directions), enabling future integration into in‐memory light sensing, neuromorphic computing, and brain‐inspired artificial vision systems.

## 3 Conclusion

In summary, we have demonstrated the synthesis and multifunctional device applications of 3L 3R‐stacked MoS_2_ nanoflakes featuring robust sliding ferroelectricity. The molecular sieve‐assisted CVD strategy enables layer modulation and 3R‐stacking uniformity, as confirmed by systematic structural and optical characterizations. The as‐grown 3L 3R‐MoS_2_ exhibits strong SHG and switchable ferroelectric polarization, confirming its noncentrosymmetric stacking configuration. When integrated into FeS‐FETs, these nanoflakes deliver outstanding electrical and optoelectronic performance, including a large memory window, ultrahigh *R*, *EQE*, and *D* ^*^, and a wide spectral response range. Furthermore, the devices exhibit key features of reconfigurable artificial optoelectronic synapses, specifically the gate‐tunable photocurrent facilitation/depression transition. This work not only advances the understanding of sliding ferroelectricity in multilayer TMDs but also establishes 3L 3R‐MoS_2_ as a promising candidate for multifunctional optoelectronic and neuromorphic technologies.

## 4 Experimental Section

### 4.1 Molecular Sieve Promoted CVD Growth of 3L 3R‐MoS_2_ Nanoflakes

The 3L 3R‐MoS_2_ nanoflakes were synthesized via a molecular sieve‐assisted CVD process within a dual‐zone tube furnace. To prepare a precise and uniform molybdenum precursor, 20 mg MoO_3_ powder was dissolved in 1 mL NH_3_·H_2_O solution (28‐30 wt.%), and subsequently 20 µL of the solution was drop‐cast onto a SiO_2_/Si piece (0.8 × 1.5 cm^2^). The SiO_2_/Si piece was heated to 70 °C to evaporate the solvent and obtain a uniform MoO_3_ coating. 30 mg molecular sieve powder was evenly distributed on top of the dried MoO_3_ film to facilitate sustained molybdenum precursor supply. A clean *c*‐plane sapphire or SiO_2_/Si substrate was placed face‐down ∼3 mm above the MoO_3_‐coated SiO_2_/Si piece to create a confined reaction space for reactants accumulation. 50 mg of sulfur powder was positioned in the upstream low‐temperature zone. During the heating process, the temperature was gradually ramped to 200 °C in the sulfur zone and 840 °C in the MoO_3_ zone over a 50 mins period. High‐purity argon (50 sccm) was used as the carrier gas throughout the process to transport the sulfur vapor and ensure an inert environment. The growth reaction was maintained at the target temperature for 10 mins, after which the furnace was naturally cooled to room temperature.

### 4.2 Transfer of 3L 3R‐MoS_2_ Nanoflakes

The as‐grown 3L 3R‐MoS_2_ nanoflakes were transferred onto carbon‐supported Cu TEM grids or SiO_2_/Si substrate for structural characterization and device fabrication. Bilayer polymers of polymethyl methacrylate (PMMA) and poly(propylene carbonate) (PPC) were sequentially spin‐coated onto the growth substrate. The polymer‐supported MoS_2_ nanoflakes were gently delaminated from the sapphire substrate in deionized water. The resulting PPC/PMMA/MoS_2_ stack was then aligned and transferred onto the target substrate. To improve interfacial contact, an additional post‐transfer baking step at 90 °C was performed. Finally, the PPC and PMMA layers were fully removed by immersing the samples in acetone for 2 h.

### 4.3 Material Characterizations

The morphology, thickness, and ferroelectric properties of the synthesized 3L 3R‐MoS_2_ nanoflakes were characterized using OM and a commercial scanning probe microscope (Dimension Icon, Bruker). A conductive SCM‐PIT‐V2 tip (spring constant of 3 N·m^−1^ and contact resonance frequency of ∼75 kHz) was employed for both imaging and electrical modulation. For domain writing, a ± 8 V DC bias was applied to the tip, while an AC voltage of 5 V was used during PFM scanning. The elemental composition and crystal structure were analyzed by XPS (SHIMADZU, AXIS SUPRA) and STEM (Spectra Ultra, Thermo Fisher Scientific) equipped with an EDS detector. Optical characterization was performed using a UV–vis‐NIR microspectrophotometer (CRAIC 20/30PV), as well as Raman and PL spectroscopies with a 532 nm excitation laser (inVia confocal, Renishaw). SHG measurements were carried out using an optical system (ScanPro Advance, Metatest) with a 1064 nm femtosecond laser (pulse width of 6 ps) as the excitation source.

### 4.4 Device Fabrication and Measurement

3L 3R‐MoS_2_ nanoflakes were transferred onto SiO_2_(300 nm)/Si substrate for device fabrication and tests. To ensure low‐resistance contacts and preserve material integrity, Au source and drain electrodes were transferred onto the MoS_2_ nanoflakes using a polymer‐assisted electrode transfer technique. Electrical and optoelectronic measurements were conducted under ambient conditions using a semiconductor probe station (CRX‐6.5K, Lake Shore Cryotronics) integrated with a semiconductor parameter analyzer (4200‐SCS, Keithley Instruments). To evaluate the photodetection and optoelectronic synapse performance, monochromatic lasers with selected wavelengths and power densities were coupled into the device via optical fibers. The photocurrent responses under varying illumination and gate bias conditions were recorded to assess broadband photoresponsivity and gate‐tunable optoelectronic behavior.

### 4.5 DFT Calculations

All calculations were performed using the density functional theory (DFT) method through Vienna Ab initio Simulation Package (VASP5.4.4). Generalized gradient approximation (GGA) of Perdew‐Burke Ernzerhof (PBE) were adopted to describe exchange correlation interaction. The ion‐electron interaction was treated using the projector augmented wave (PAW) technique. The plane‐wave cutoff energy of 450 eV was employed.

## Conflicts of Interest

The authors declare no conflicts of interest.

## Supporting information




**Supporting File**: advs73836‐sup‐0001‐SuppMat.docx.

## Data Availability

The data that support the findings of this study are available from the corresponding author upon reasonable request.
