# Supplementary material for: Molecular Sieve Promoted Growth of Ferroelectric Trilayer 3R‐MoS2 for Polarization‐Dependent Reconfigurable Optoelectronic Synapses
Source: Adv Sci (Weinh). 2026 Jan 20;13(17):e24333. doi: 10.1002/advs.202524333 (PMC13042948; doi:10.1002/advs.202524333)
Supplement: Supplementary file 1 — Supporting File: advs73836‐sup‐0001‐SuppMat.docx. [file ADVS-13-e24333-s001.docx]

Supporting Information

Molecular Sieve Promoted Growth of Ferroelectric Trilayer 3R-MoS_2_ for Polarization-Dependent Reconfigurable Optoelectronic Synapses

Qichao Xue, Jincheng Zhang, Yuying Wang, Yu Wang, Kuiwei Li, Yuxuan Chen, Fang Zhong, Qing Li, Ning Zhou, Chenying Yang, Yuchuan Shao, and Tao Liang*


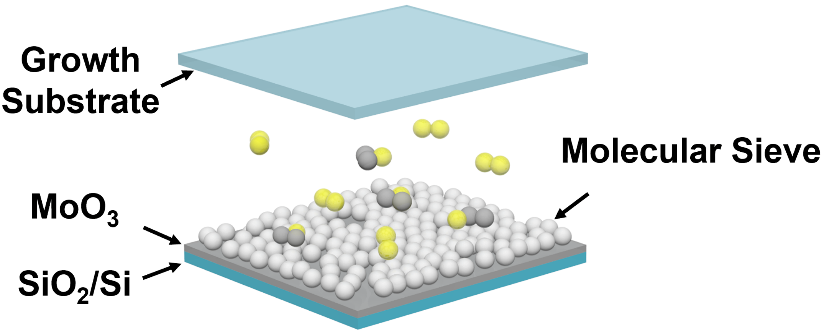


**Figure S1.** Schematic of the molecular sieve promoted CVD growth of 3L 3R-MoS_2_ nanoflakes. The grey, yellow, and white spheres represent the molybdenum species, sulfur species, and the molecular sieve powders, respectively.


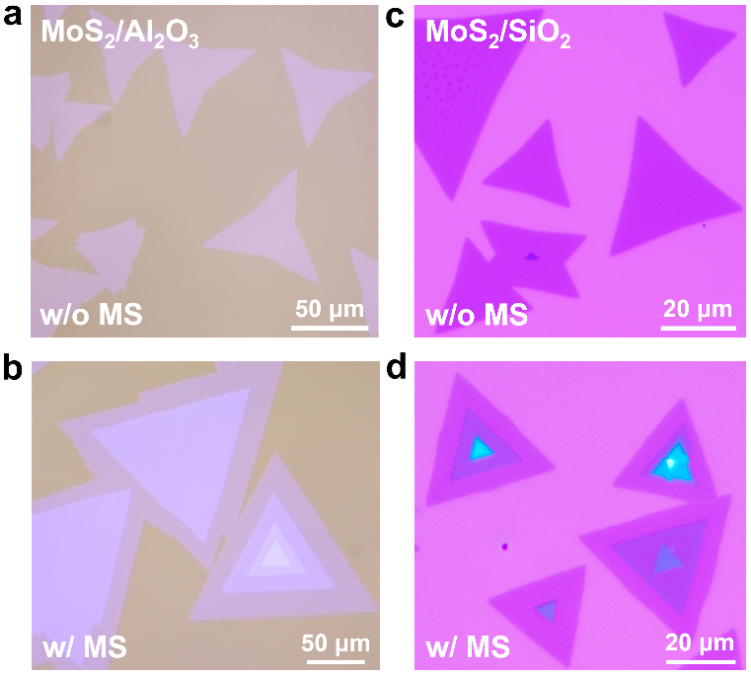


**Figure S2.** OM images of the growth results comparison. (a, b) MoS_2_ growth on sapphire (Al_2_O_3_) substrate (a) without (w/o) and (b) with (w/) the assistance of molecular sieve (MS). (c, d) MoS_2_ growth on SiO_2_/Si substrate (c) without and (d) with the assistance of molecular sieve.


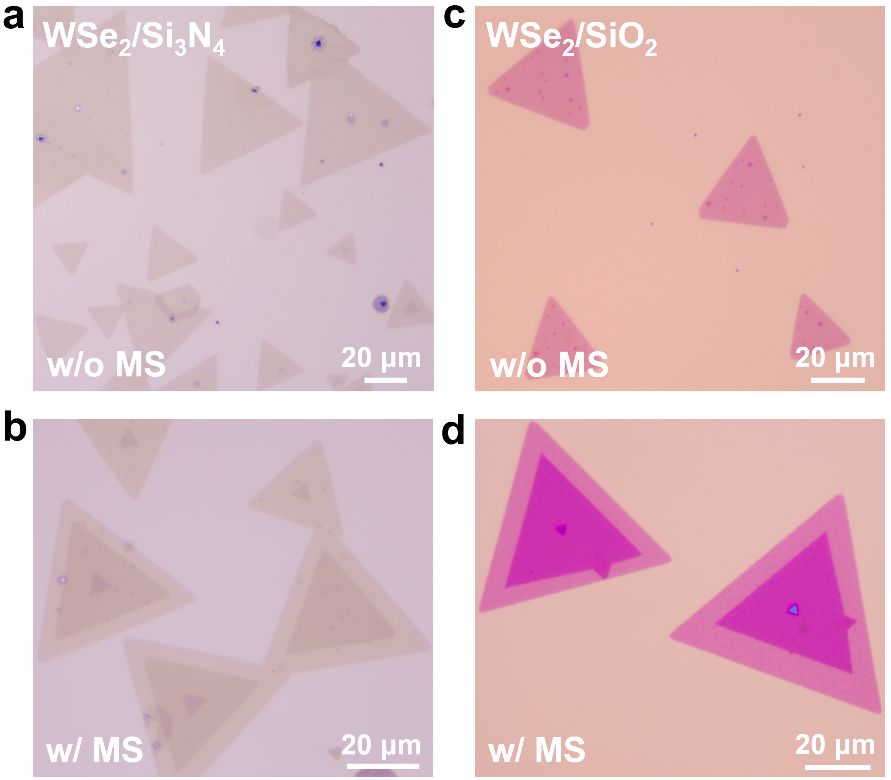


**Figure S3.** OM images of the growth results comparison. (a, b) WSe_2_ growth on Si_3_N_4_ substrate (a) without and (b) with the assistance of molecular sieve. (c, d) WSe_2_ growth on SiO_2_/Si substrate (c) without and (d) with the assistance of molecular sieve.


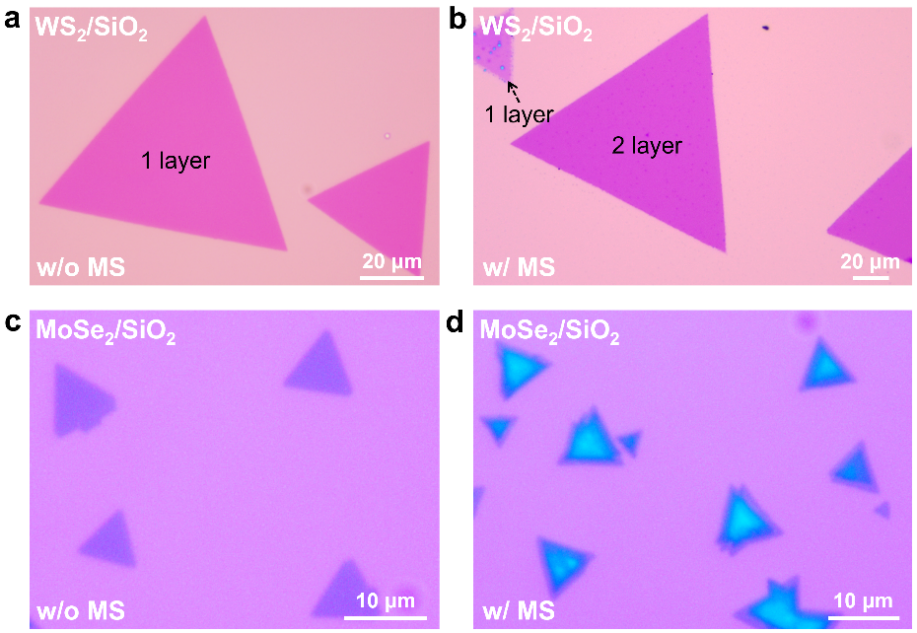


**Figure S4.** OM images of the growth results comparison. (a, b) WS_2_ growth on SiO_2_/Si substrate (a) without and (b) with the assistance of molecular sieve. (c, d) MoSe_2_ growth on SiO_2_/Si substrate (c) without and (d) with the assistance of molecular sieve.


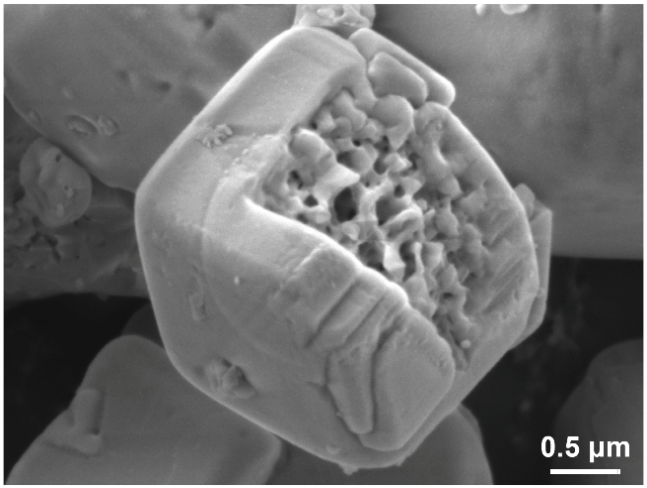


**Figure S5.** SEM image of the porous molecular sieve powders.


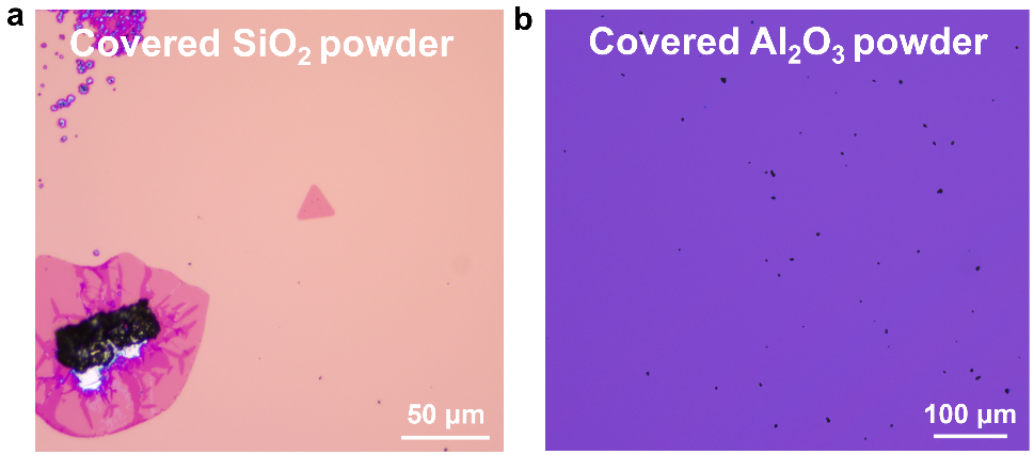


**Figure S6.** OM image of the MoS_2_ growth results when porous SiO_2_ and Al_2_O_3_ powders were covered onto the molybdenum precursors to replace the molecular sieve powders.


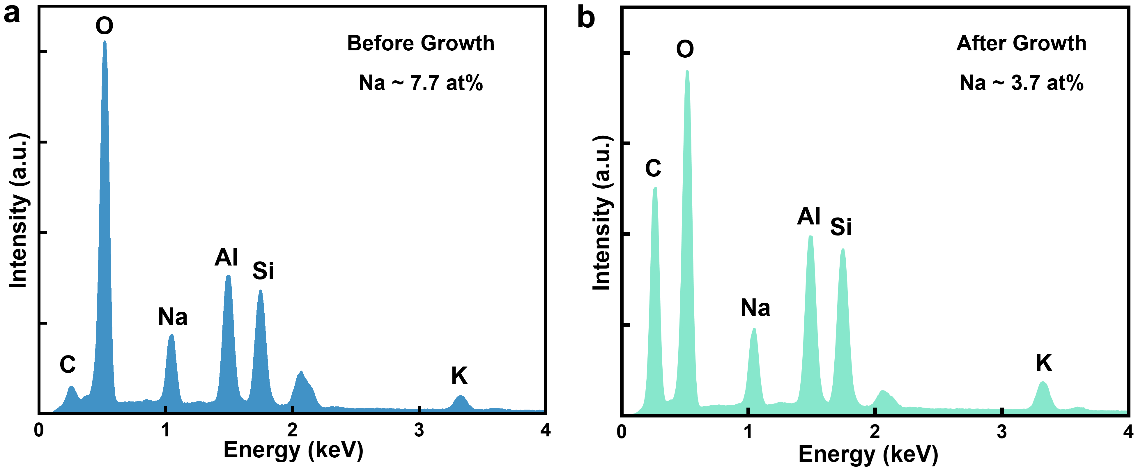


**Figure S7.** SEM-EDS results of the molecular sieve powders (a) before and (b) after the CVD growth.


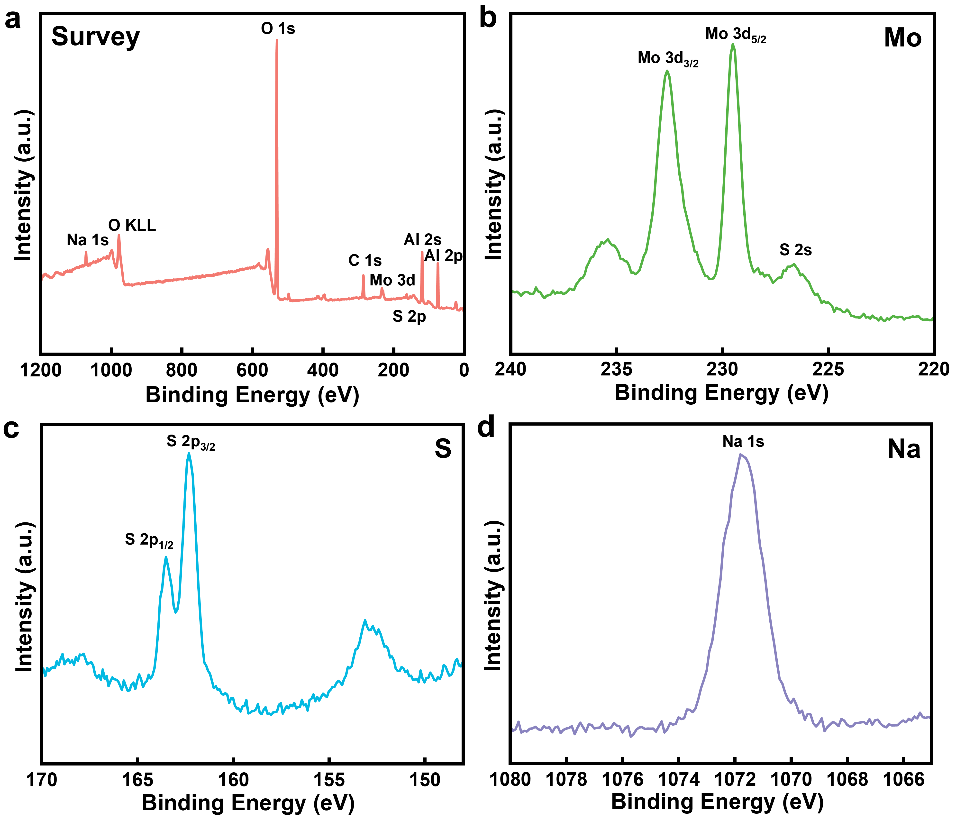


**Figure S8.** XPS results of the substrate surface after the MoS_2_ growth. (a) Survey spectrum. (b, c) Mo 3d and S 2p spectra. (d) Na 1s spectrum, highlight the presence of Na elements on the substrate surface after the CVD growth.


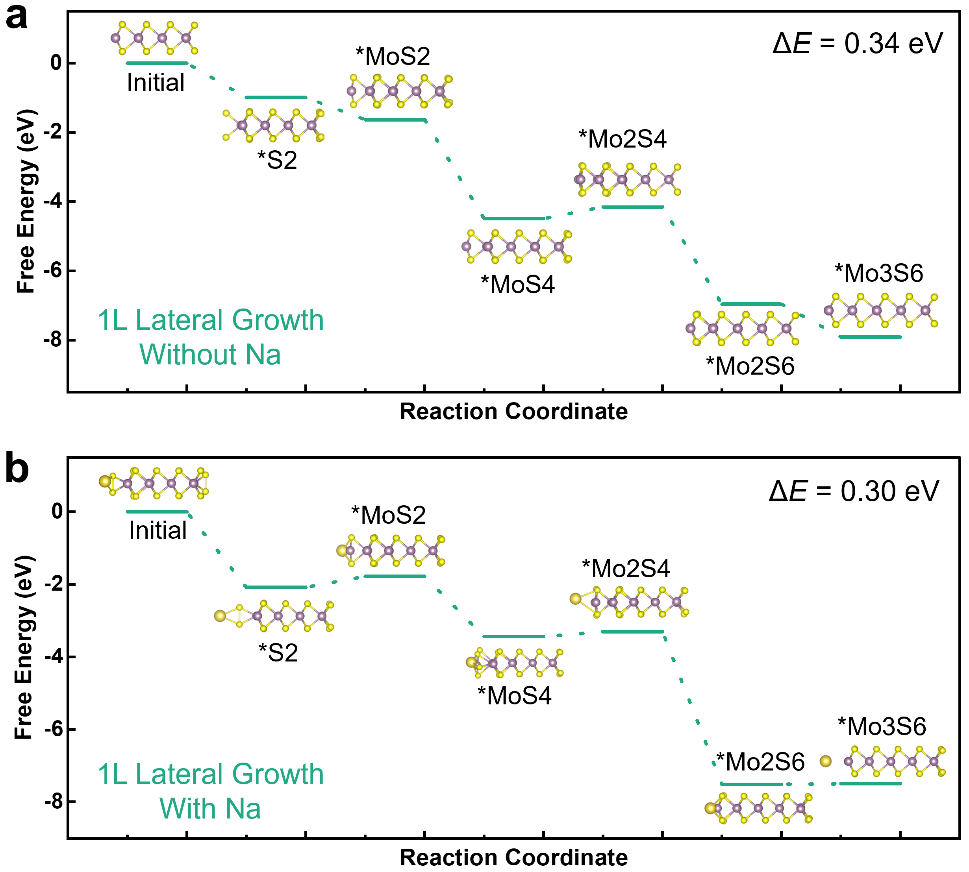


**Figure S9.** DFT calculation of the monolayer MoS_2_ growth process (a) without and (b) with the alkali Na element.


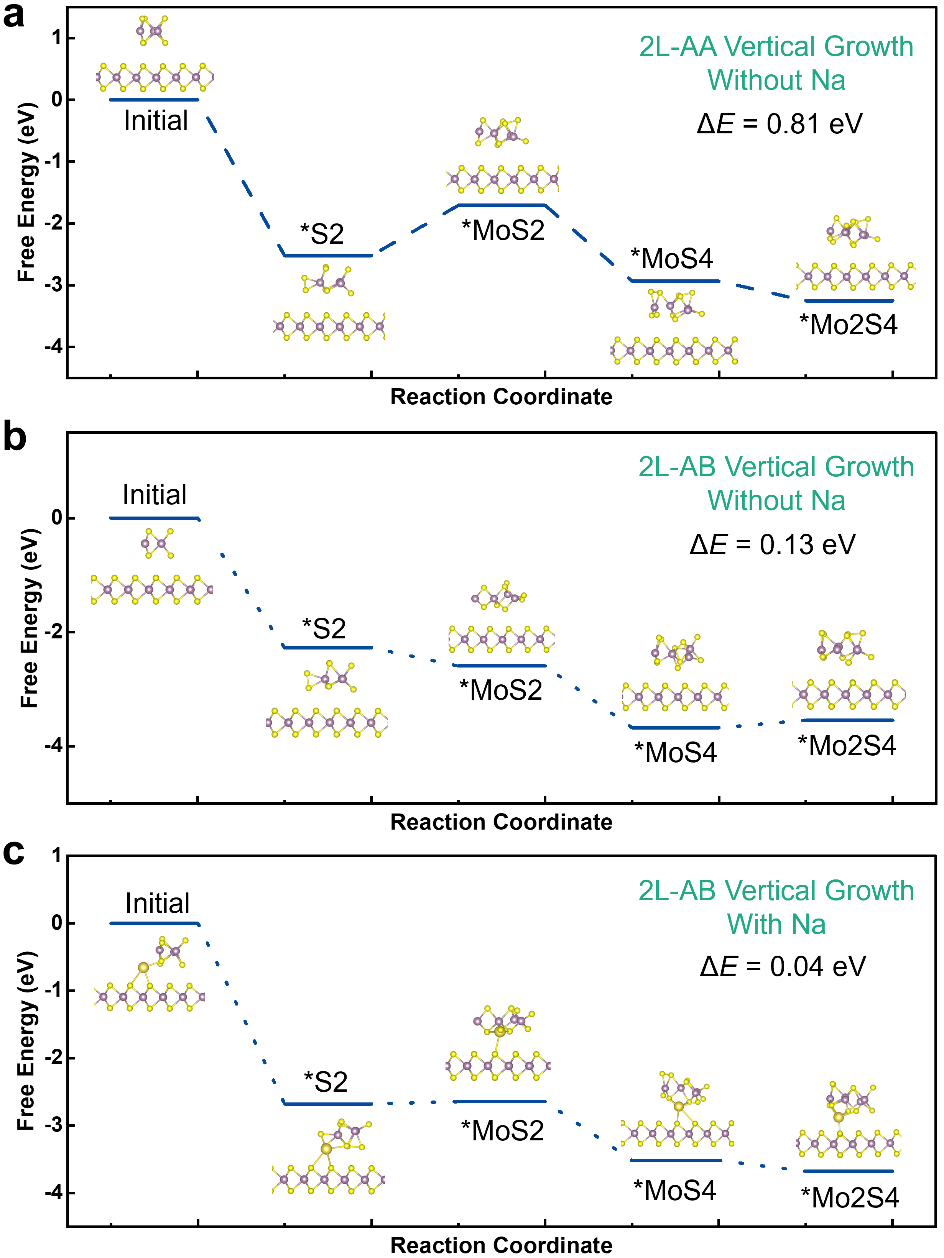


**Figure S10.** DFT calculation of the growth process of (a) 2L 3R-stacked MoS_2_ without Na adsorption. (b, c) 2L 2H-stacked MoS_2_ (b) without and (c) with Na adsorption.


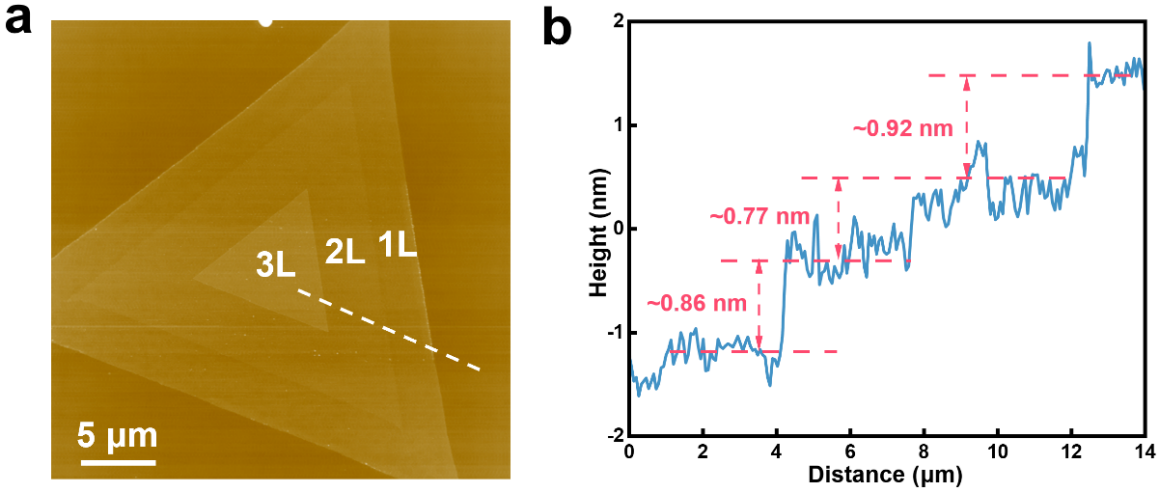


**Figure S11.** AFM characterization of one 3L 3R-MoS_2_ nanoflake. (a) Scanning AFM height image of a 3L 3R-stacked MoS_2_. (b) The height profile along the dashed line in (a) showing the stepped 3L nature of the MoS_2_ nanoflake.


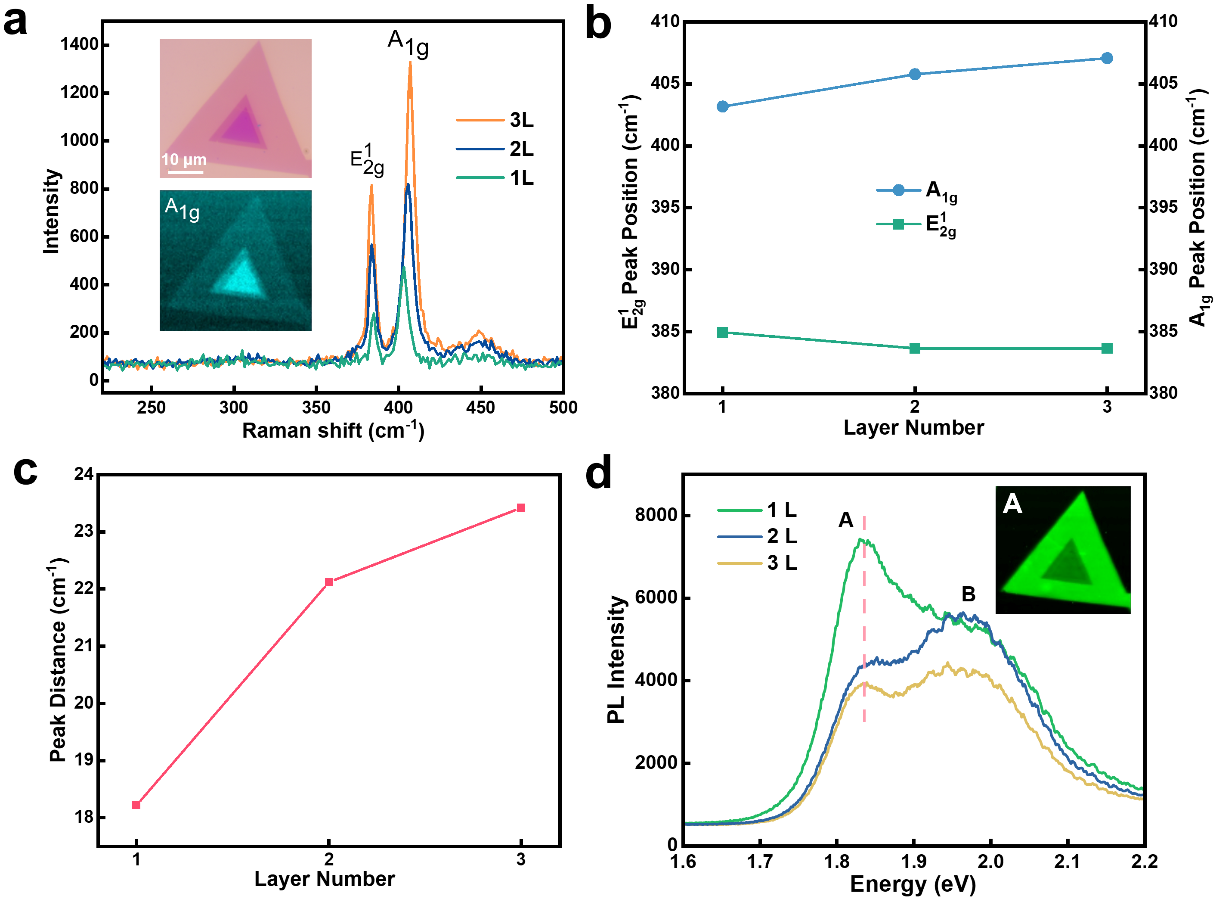


**Figure S12.** Raman and PL characterizations of the 3L 3R-MoS_2_ nanoflakes. (a) Raman spectra acquired from the 1L, 2L, and 3L regions of the same nanoflake. Insets show the OM image of one MoS_2_ nanoflake and its Raman A_1g_ peak intensity mapping. (b) The position of the Raman peaks changes as the 3R-stacked MoS_2_ layer number increases from 1 to 3. (c) The peak distance increases with increasing layer number. (d) PL spectra acquired from the 1L, 2L, and 3L regions of the nanoflake. Inset shows the excitonic A peak intensity mapping.

Notes:

The layer-dependent optical properties of the 3L 3R-MoS_2_ nanoflake were investigated (OM image shown in the inset of Figure S12a). As shown in Figure S12a, the characteristic *E*^1^_2g_ and *A*_1g_ phonon modes of MoS_2_ are clearly resolved across all thicknesses, with their intensity increasing notably from 1L to 3L. Raman intensity mapping (inset of Figure S12a) further confirms the spatial uniformity of the multilayer stacking. Additionally, a monotonic increase in peak separation between *E*^1^_2g_ and *A*_1g_ modes is observed with increasing layer number, from 18.2 cm^-1^ in 1L to 23.4 cm^-1^ in 3L (Figure S12b and S12c). This change primarily arises from a pronounced blueshift of the *A*_1g_ peak and a slight redshift of the *E*^1^_2g_ peak. The *A*_1g_ mode, associated with out-of-plane atomic vibrations influenced by vdW restoring forces, generally shifts to higher frequency with enhanced interlayer coupling. In contrast, the *E*^1^_2g_ mode reflects the in-plane atomic vibration and exhibits slight frequency change due to long-range Coulombic interactions induced dielectric screening effect.^[1]^ As such, the progressive blueshift of the *A*_1g_ mode as layer increases suggests strong interlayer interactions in the 3R-stacking configuration.

Photoluminescence (PL) spectra of 1-3L 3R-MoS_2_ are presented in Figure S12d, offering insights into the excitonic transitions and layer-dependent band structure. For the 1L region, distinct PL peaks are observed at 1.80 eV and 1.97 eV, corresponding to the A and B exciton transitions, respectively. These transitions originate from the spin-orbit coupling (SOC) induced valance band splitting (VBS) at the K point of the Brillouin zone. The A exciton peak remains nearly constant in the 2L and 3L regions, consistent with earlier reports.^[2]^ The PL intensity mapping of the MoS_2_ nanoflake (inset in Figure S12d) reveals a clear spatial correlation between 3R-MoS_2_ layers and PL emission. While the outer 1L region exhibits strong PL, the inner 2L and 3L regions display relatively weaker yet uniform PL intensity, further confirming the spatially resolved layer configuration and high crystalline quality.


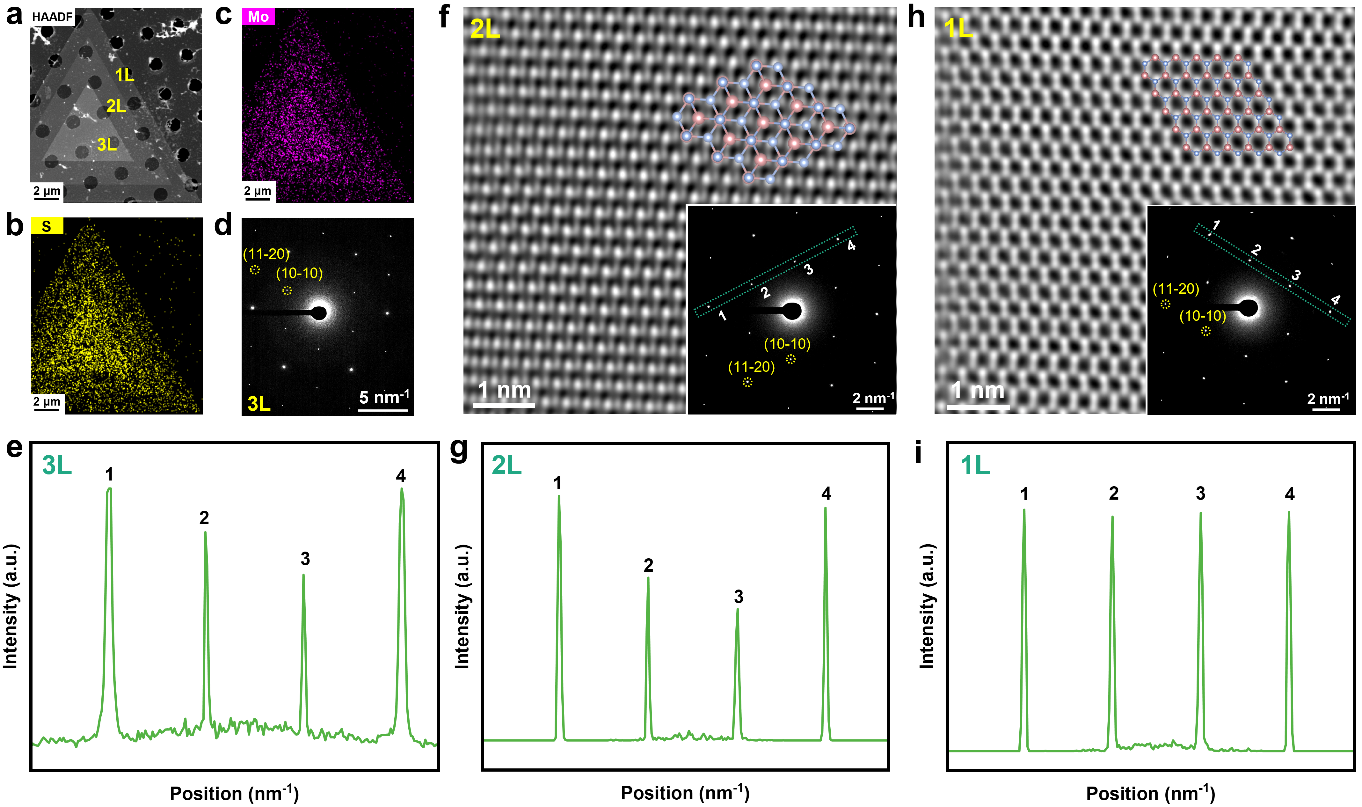


**Figure S13.** STEM characterizations to confirm the 3R-stacked configuration in the 3L 3R-MoS_2_ nanoflake. (a) Low-magnification TEM image of a typically transferred 3R-stacked 3L MoS_2_ nanoflake. (b, c) EDS mappings of the S and Mo elements taken from the nanoflake in (a). (d) The SAED pattern taken from the 3L region of the nanoflake in (a). (e) The line intensity profile taken along four diffraction spots from the SAED pattern of 3L region. (f) STEM image of the 2L region in (a). The inset shows the corresponding SAED pattern. (g) The line intensity profile taken along the four diffraction spots in the SAED pattern inset in (f). (h) STEM image of 1L MoS_2_. The inset shows the corresponding SAED pattern. (i) The line intensity profile taken from the dashed rectangle in the SAED pattern inset in (h). The overlaid atomic structures in (f) and (h) show the atoms arrangement in 3R-stacked 2L and 1L MoS_2_, respectively..

Notes:

The atomic structure and stacking configuration of the MoS_2_ nanoflakes were examined using transmission electron microscopy (TEM). A high-angle annular dark-field (HAADF) TEM image of a 3L 3R-MoS_2_ nanoflake transferred onto a carbon-supported Cu grid is shown in Figure S13a, revealing a compact and well-preserved layer-by-layer structure. Elemental mapping via energy-dispersive X-ray spectroscopy (EDS) confirms the homogeneous distribution of sulfur and molybdenum atoms across the nanoflake (Figure S13b and S13c), with signal intensity increasing from the 1L edge to the central 3L region, consistent with increasing thickness. The selected-area electron diffraction (SAED) pattern (Figure S13d) acquired from the central 3L region displays a single hexagonal diffraction pattern, indicating the single-crystalline nature of the 3L 3R-MoS_2_. Scanning transmission electron microscope (STEM) imaging of the 3L region (Figure 1d) reveals a well-ordered hexagonal arrangement of overlapping molybdenum and sulfur atomic columns, consistent with the structural model illustrated in Figure 1a. Comparative STEM images from the 2L and 1L regions are also captured and presented (Figure S13f and S13h). The 3R-stacked 2L region exhibits atomic columns at both the vertices and center of the hexagonal lattice, whereas the 1L region shows alternating molybdenum and sulfur atoms only at the corners, highlighting the evolution of stacking order. These structural assignments are further corroborated by SAED patterns from the corresponding regions (insets of Figure S13f and S13h) as well as quantitative analysis of the diffraction spot intensities (Figure S13e, S13g, S13i). Specifically, in the 2L 3R region, the averaged intensity profile from the four marked diffraction spots reveals that the {11-20} lattice planes exhibit approximately threefold higher intensity than the {10-10} planes (Figure S13g). In contrast, the intensity of these planes is nearly identical in the 1L region (Figure S13i), reflecting the symmetric sublattice structure of 1L MoS_2_. These results collectively confirm the high crystallinity and well-defined 3R-stacking configuration of the 3L MoS_2_ nanoflakes synthesized via the molecular sieve-assisted CVD process.


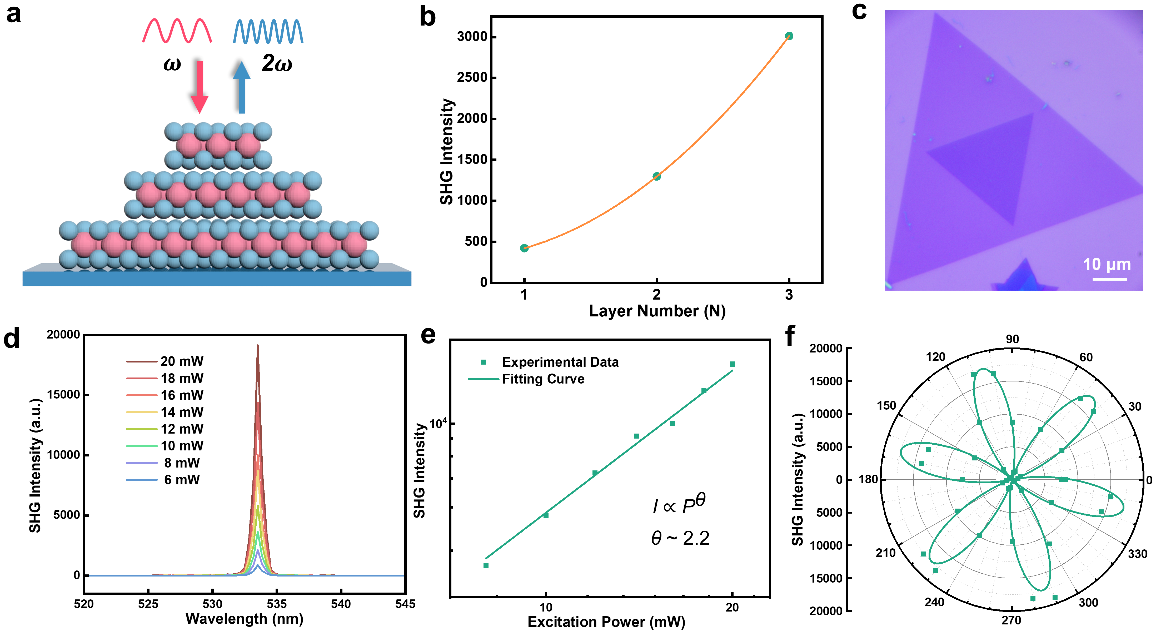


**Figure S14.** SHG characterizations of the 3R-stacked 3L MoS_2_ nanoflakes under a 1064 nm excitation laser. (a) Schematic diagram of the SHG measurement. (b) The fitted quadratic dependence of SHG intensity on the layer number in 3R-stacked MoS_2_ nanoflakes. (c) OM image of the 2L MoS_2_ nanoflake with 2H-stacked configuration for SHG intensity comparison. (d, e) SHG intensity versus excitation power and the fitted relationship for 3L region of the 3R-stacked nanoflake. (f) Polarization dependence of the SHG intensity, exhibiting a 6-fold rotational symmetry.


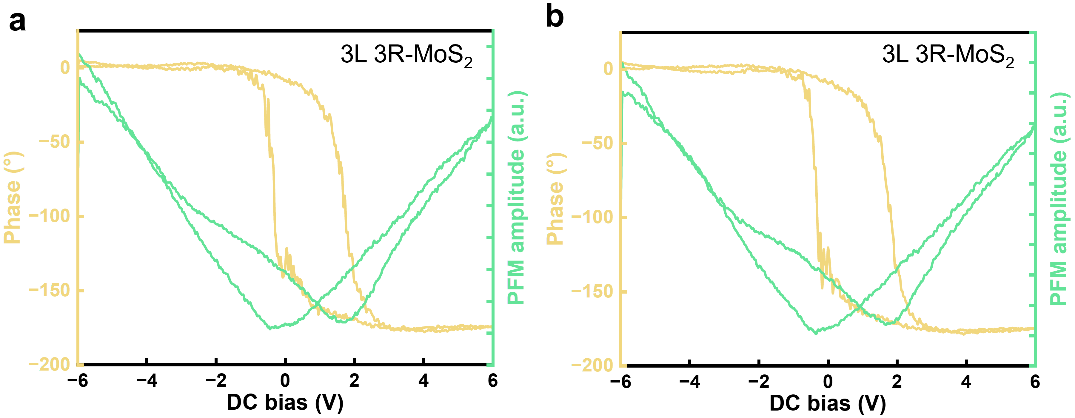


**Figure S15.** PFM amplitude and phase hysteresis loops acquired from other two different 3L 3R-MoS_2_ nanoflakes.


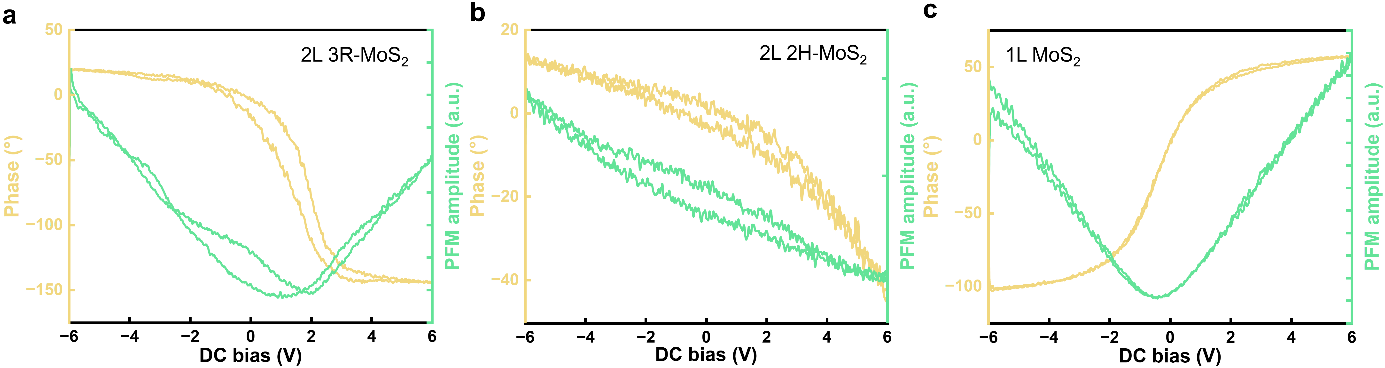


**Figure S16.** PFM amplitude and phase hysteresis loops acquired from (a) 2L 3R-MoS_2_ nanoflake, (b) 2L 2H-MoS_2_ nanoflake, and (c) 1L MoS_2_ nanoflake.


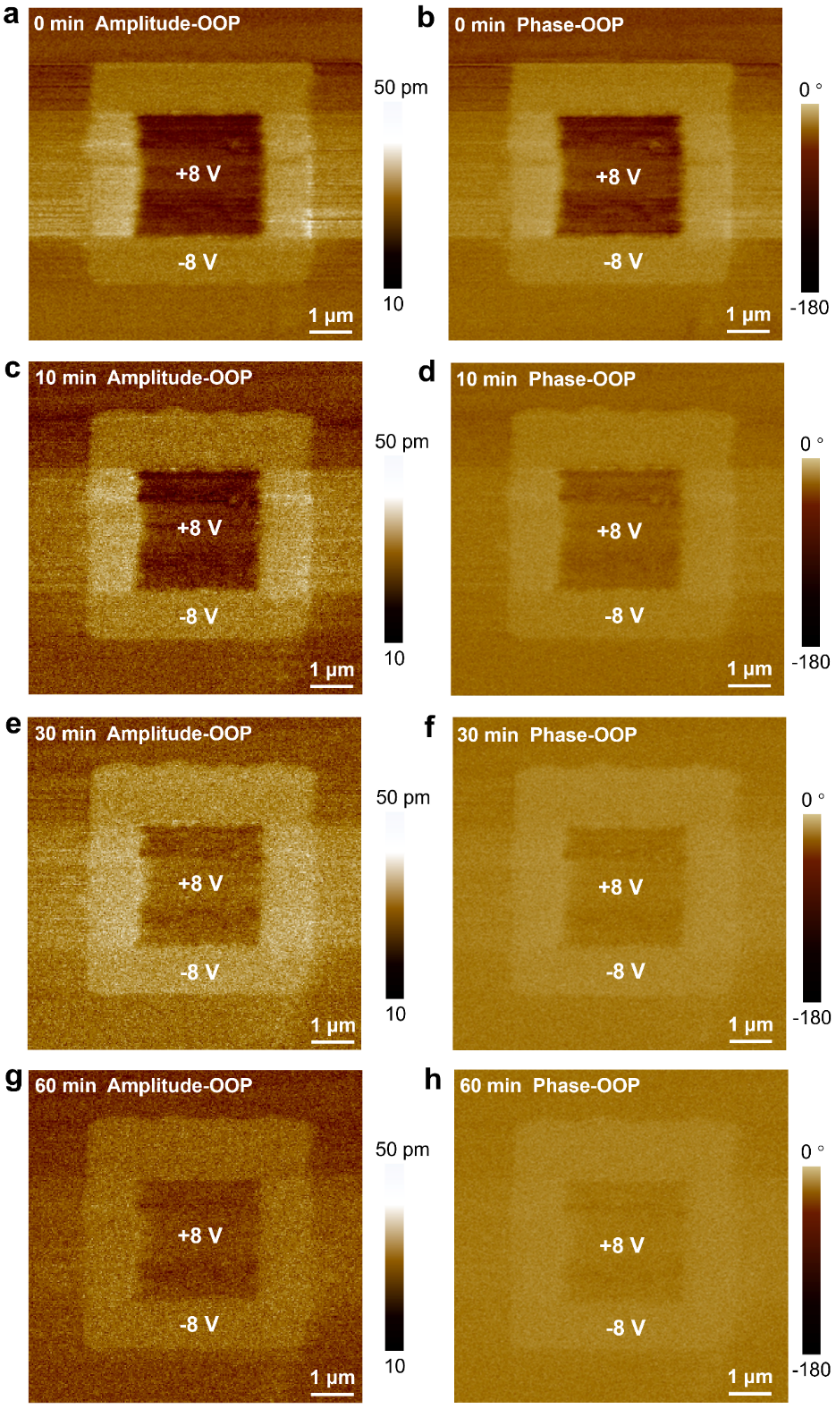


**Figure S17.** (a, b) The out-of-plane PFM amplitude and phase images acquired after writing with +8 V and -8 V DC bias in the box pattern. The PFM images from the same area were acquired again after (c, d) 10 min, (e, f) 30 min, (g, h) 60 min retention time.


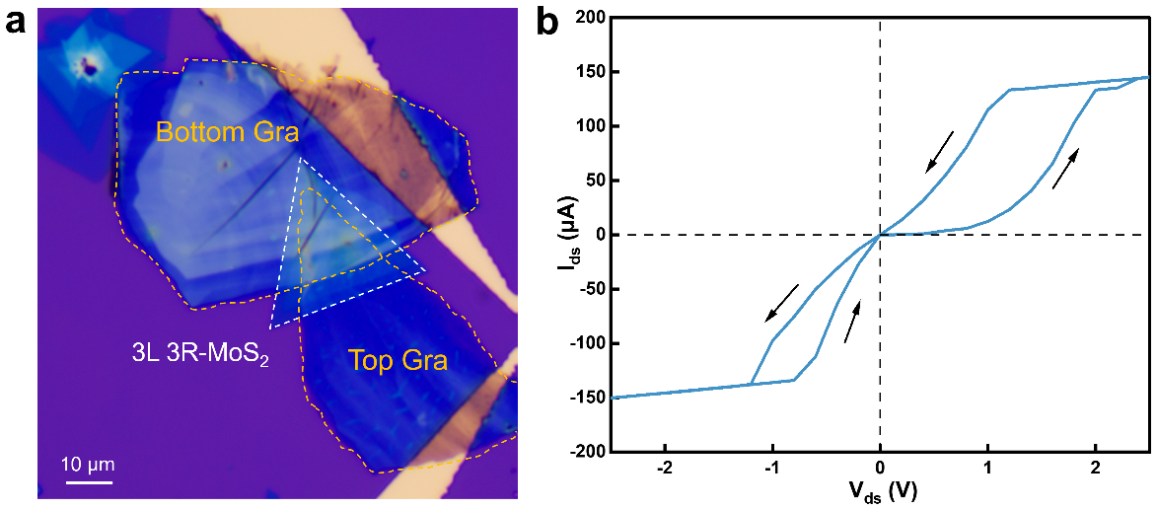


**Figure S18.** (a) OM image of the vertically-stacked bottom graphene (Gra)/3L 3R-MoS_2_/top graphene device. (b) The cyclic *I*_ds_-*V*_ds_ sweep curve for the vertical device.


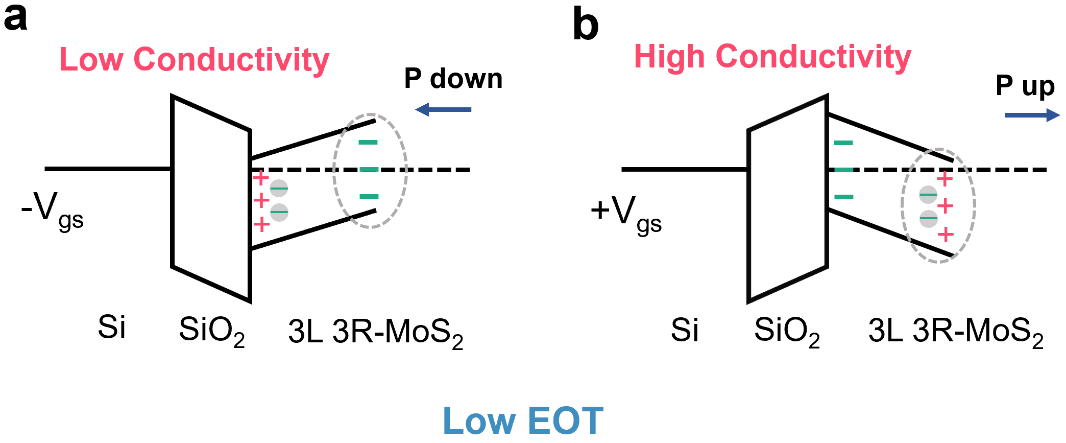


**Figure S19.** The polarization states and free carrier accumulation/depletion under positive and negative gate voltage under a low EOT condition.


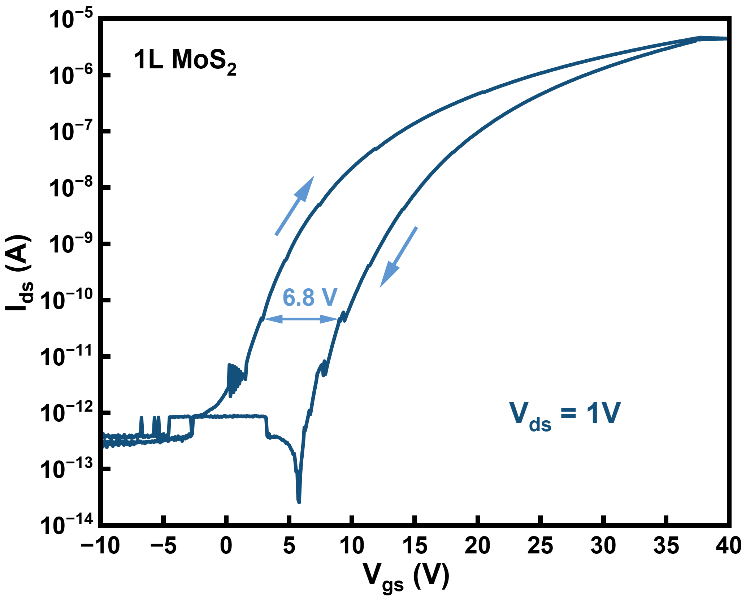


**Figure S20.** The transfer curve for the FET device based on non-ferroelectric 1L MoS_2_ nanoflake.

Notes:

The hysteresis direction in the FET *I*_ds_-*V*_gs_ transfer characteristics provides important information regarding the dominant mechanism, either governed by interfacial charge trapping/detrapping or by ferroelectric polarization switching.

In conventional n-type MoS_2_ FETs employing SiO_2_ gate dielectrics, hysteresis is generally dominated by charge trapping at the MoS_2_/SiO_2_ interface or by the adsorption of polar molecules such as H_2_O and O_2_ on the MoS_2_ surface. These extrinsic effects typically induce clockwise hysteresis, as widely documented.^[3,4]^ To verify this, we fabricated control monolayer MoS_2_ FETs (which lack intrinsic ferroelectricity due to mirror symmetry) on SiO_2_/Si substrates and measured them under identical conditions (ambient conditions, ~ 50% relative humidity). The control device exhibits a distinct clockwise hysteresis with a memory window of ~ 6.8 V (Figure S20), consistent with previous reports. This behavior originates from the gradual accumulation of trapped electrons at the interface during forward gate sweeping, which partially screens the gate electric field. Upon backward sweeping, these trapped charges are released slowly, resulting in a positive threshold voltage shift and hence a clockwise hysteresis loop.

By contrast, conventional ferroelectric FETs (FeFETs) that incorporate ferroelectric dielectrics such as P(VDF-TrFE) or Hf_1-x_Zr_x_O_2_ typically display anticlockwise hysteresis.^[5,6]^ In these devices, polarization switching in the ferroelectric dielectric modulates the semiconductor surface potential, with polarization orientation controlling carrier accumulation or depletion during the gate sweeps. However, it is worth noting that clockwise hysteresis has also been reported in FeFETs incorporating Pb(Zr,Ti)O_3_ (PZT) or MOFs,^[7,8]^ where interfacial charge trapping and partial polarization screening compete with the intrinsic ferroelectric switching.

Our devices belong to a distinct class of ferroelectric semiconductor FETs (FeS-FETs), where the active channel itself (3L 3R-MoS_2_) simultaneously exhibits semiconducting transport and spontaneous polarization. The direction of hysteresis in FeS-FETs depends strongly on the strength of the gate-induced vertical electric field across the ferroelectric semiconductor. In our device, the 3L MoS_2_ nanoflakes are grown on a 300 nm SiO_2_ dielectric layer, which provides a low gate capacitance and consequently a weak vertical electric field. Under such conditions, the gate bias mainly modulates the bottom interface of the MoS_2_ channel rather than the entire nanoflake. When a negative *V*_gs_ is applied, positive polarization-bound charges accumulate at the bottom interface and negative bound charges at the top surface (Figure 2c and 2d). This induces downward band bending and attracts electrons to the bottom surface, thereby enhancing channel conductance. Conversely, under positive *V*_gs_, negative polarization charges at the bottom repel electrons, reducing conductance (Figure 2e and 2f). This asymmetric conductance modulation produces a clockwise hysteresis loop, in excellent agreement with our experimental observations.

When the dielectric layer is much thinner or possesses a higher dielectric constant (i.e., lower equivalent oxide thickness, EOT), the gate field becomes sufficiently strong to penetrate through the entire 3L MoS_2_ channel, enabling complete polarization switching. In this regime, the channel conductance is dominated by the top surface of the MoS_2_ layer. A negative *V*_gs_ drives downward polarization and electron depletion at the top surface (low conductance, Figure S19a), while a positive *V*_gs_ reverses the polarization upward, inducing electron accumulation (high conductance, Figure S19b), yielding an anticlockwise hysteresis loop.

Therefore, the observed clockwise hysteresis in our FeS-FETs arises from the combined effects of: (i) partial polarization switching confined near the bottom interface due to the weak gate field, and (ii) charge trapping and detrapping at the MoS_2_ surface. Notably, the memory window of our 3L 3R-MoS_2_ FeS-FETs (~ 14 V) is significantly larger than that of the control monolayer MoS_2_ FETs (~6.8 V), confirming that ferroelectric polarization contributes substantially in addition to interfacial charge trapping. These results demonstrate that the clockwise hysteresis observed in our devices originates from a cooperative interplay between sliding ferroelectricity and interface effects.


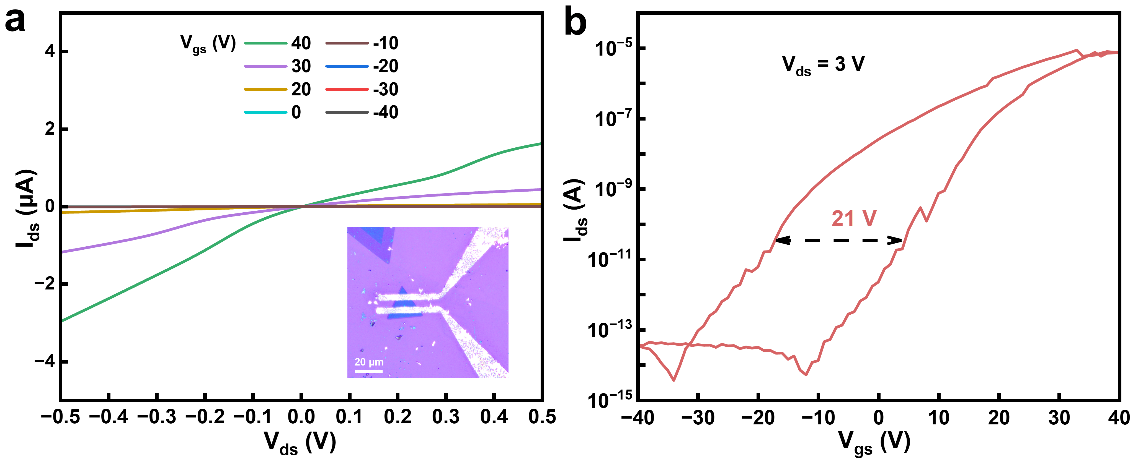


**Figure S21.** The output and transfer curves obtained from 3L 3R-MoS_2_ nanoflakes with a uniform morphology.


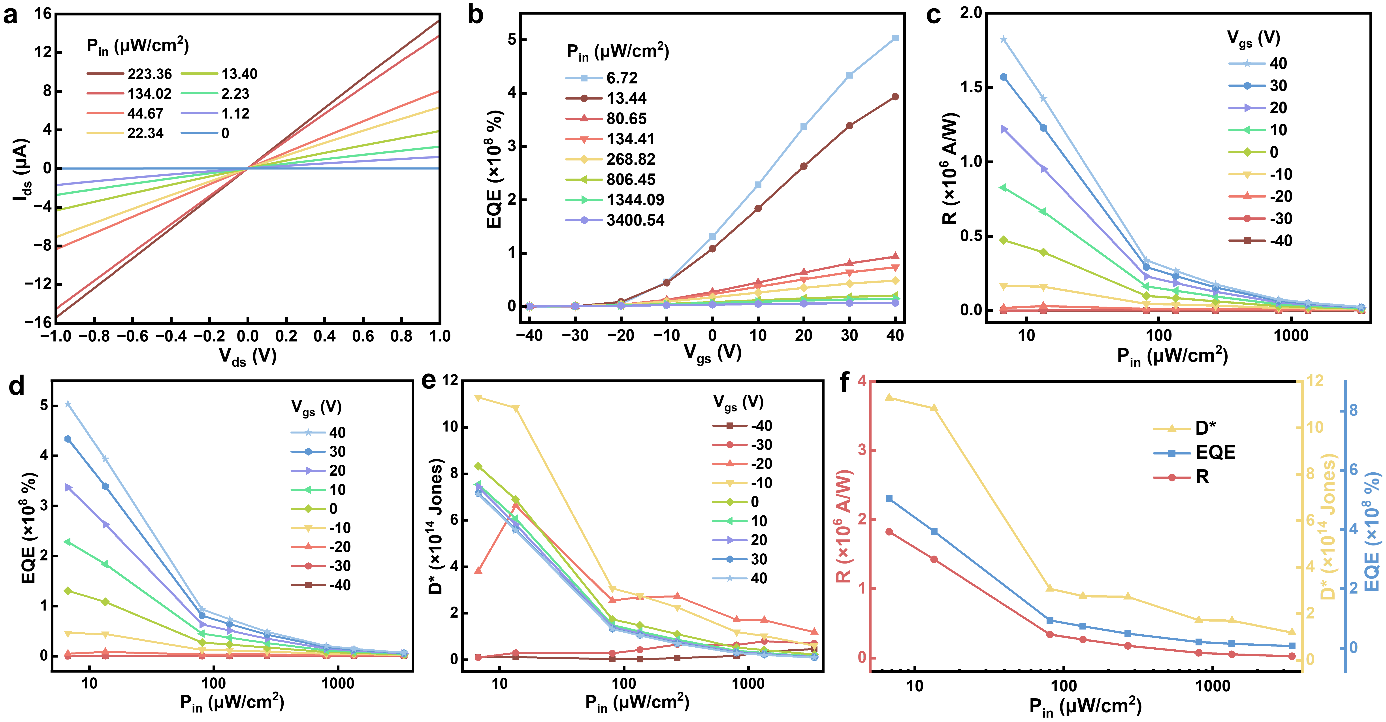


**Figure S22.** The photodetection performance under 450 nm laser illumination. (a) The output characteristics of the 3L 3R-MoS_2_ phototransistor under increasing laser power densities. (b) The calculated *EQE* as a function of *V*_gs_ under increasing laser power densities. (c-e) The calculated (c) *R*, (d) *EQE*, and (e) *D*^*^ as a function of increasing incident power densities under different *V*_gs_. (f) A general decreasing trend of *R*, *EQE*, and *D** is observed as the power density of the 450 nm incident laser is increased.


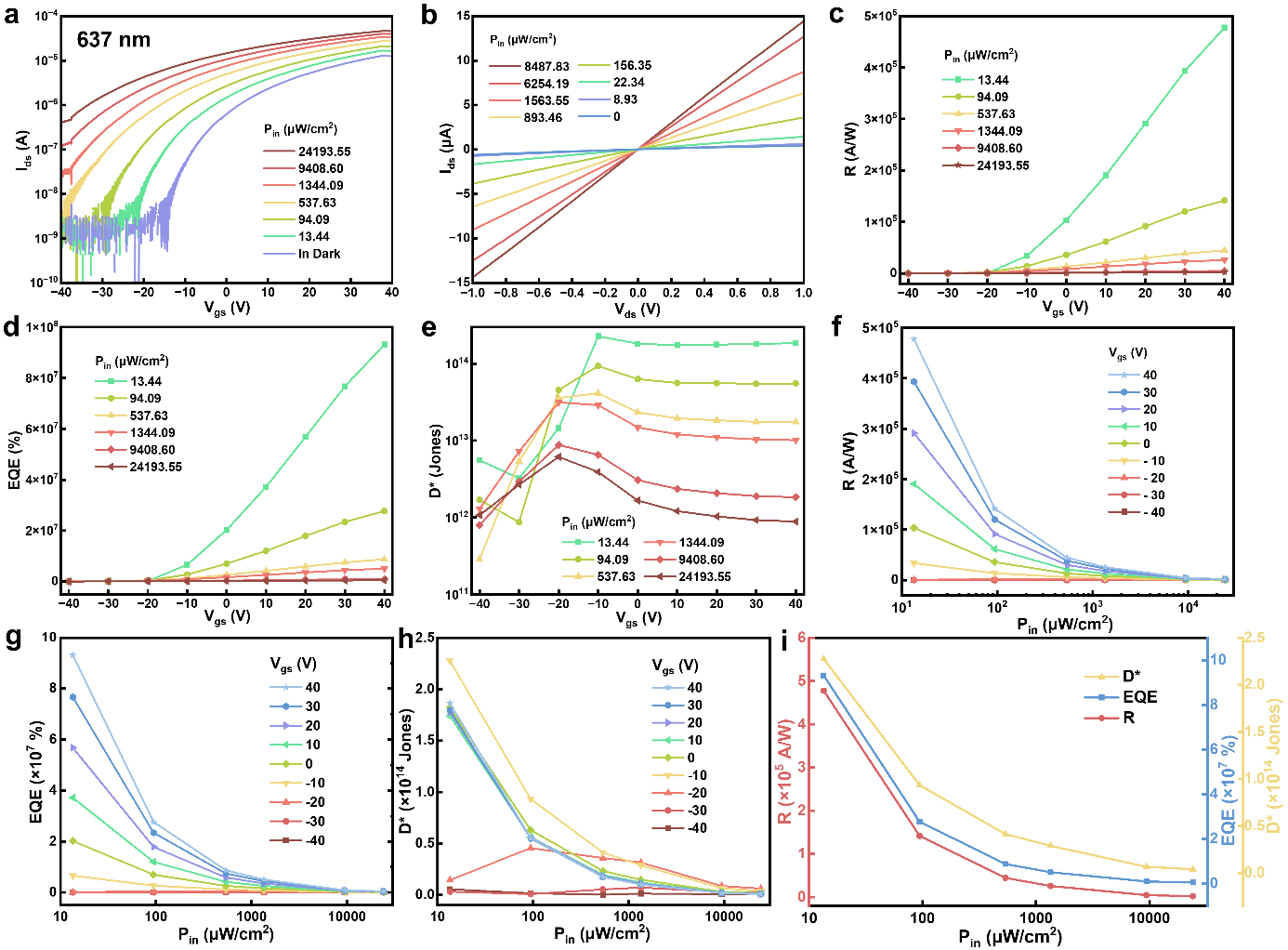


**Figure S23.** The 3L 3R-MoS_2_ photodetector performance under 637 nm laser illumination. (a) The transfer characteristics of the phototransistor illuminated with increasing incident power densities at *V*_ds_ = 1 V. (b) The output characteristics of the phototransistor under increasing laser power densities at *V*_gs_ = 0 V. (c-e) The calculated (c) *R*, (d) *EQE*, and (e) *D*^*^ from (a) as a function of *V*_gs_ under increasing incident power densities. (f-h) The calculated (f) *R*, (g) *EQE*, and (h) *D*^*^ as a function of increasing incident power densities under diverse *V*_gs_. (i) A general decreasing trend of *R*, *EQE*, and *D*^*^ is observed as the power density of the 637 nm incident laser is increased.


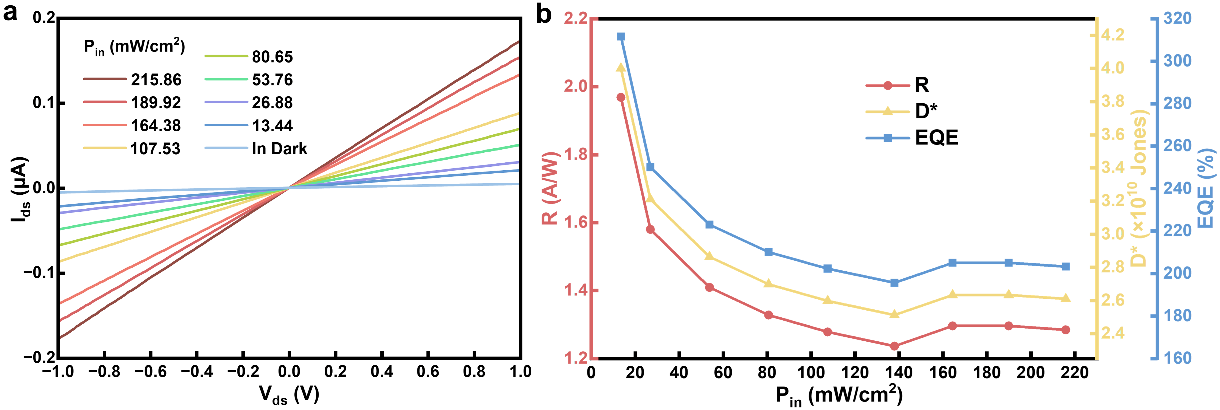


**Figure S24.** The 3L 3R-MoS_2_ photodetector performance under 785 nm laser illumination. (a) The output characteristics of the phototransistor under increasing laser power densities at *V*_gs_ = 0 V. (b) The calculated *R*, *EQE*, and *D*^*^ as the power density of the 785 nm incident laser is increased.


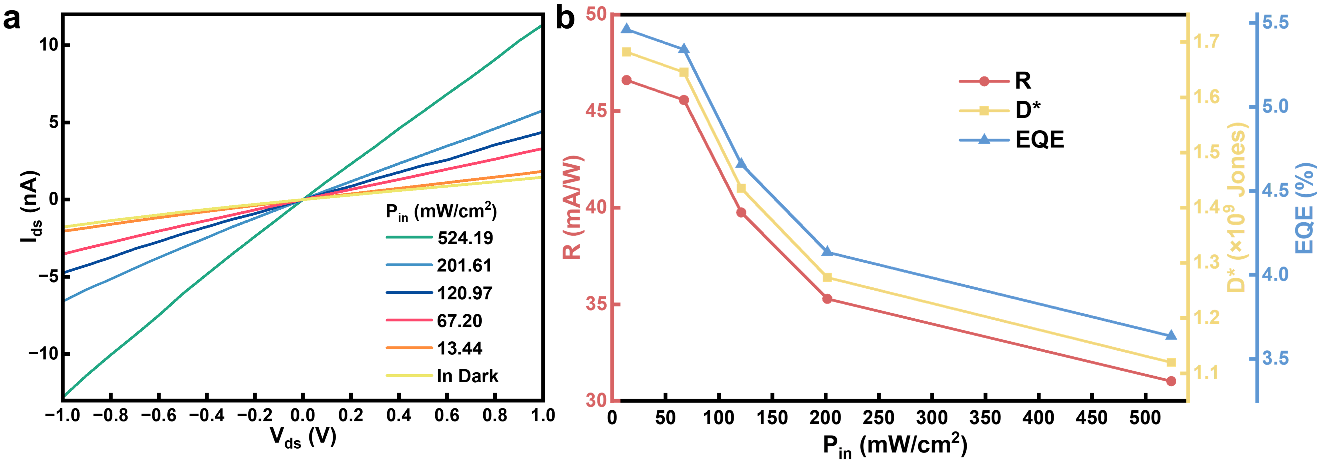


**Figure S25.** The 3L 3R-MoS_2_ photodetector performance under 1060 nm laser illumination. (a) The output characteristics of the phototransistor under increasing laser power densities at *V*_gs_ = 0 V. (b) The calculated *R*, *EQE*, and *D*^*^ as the power density of the 1060 nm incident laser is increased.


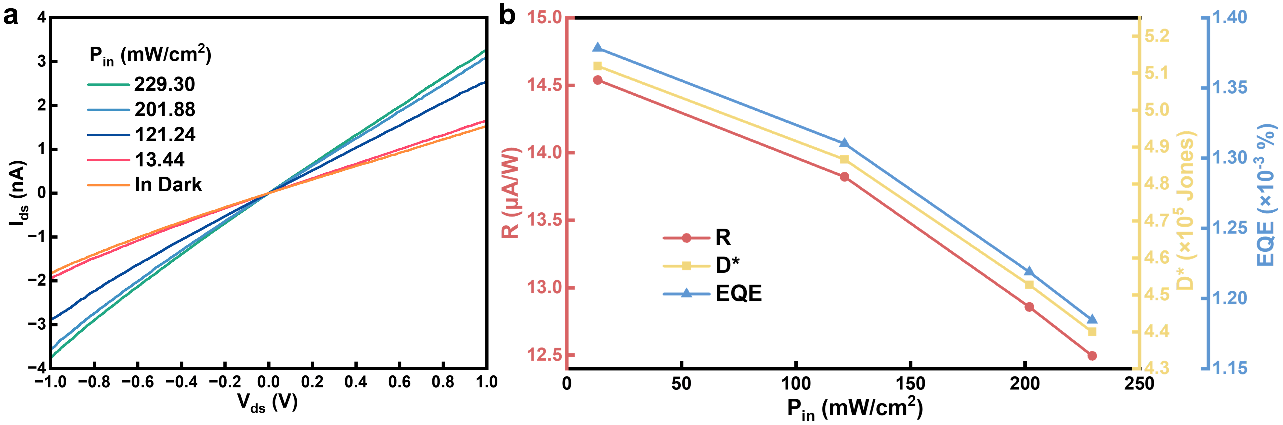


**Figure S26.** The 3L 3R-MoS_2_ photodetector performance under 1310 nm laser illumination. (a) The output characteristics of the phototransistor under increasing laser power densities at *V*_gs_ = 0 V. (b) The calculated *R*, *EQE*, and *D*^*^ as the power density of the 1310 nm incident laser is increased.


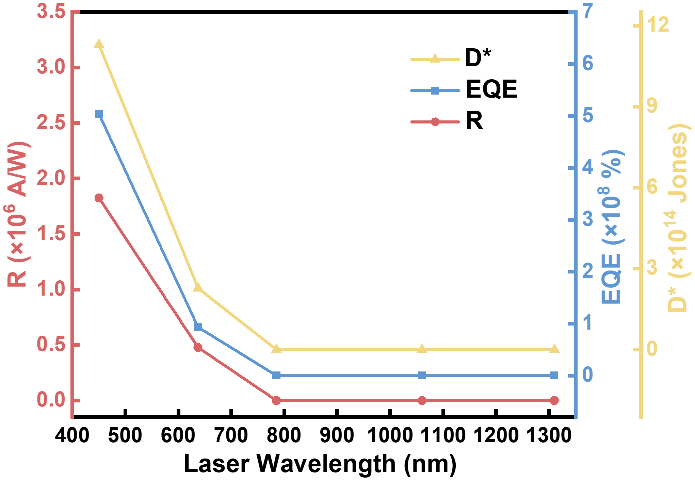


**Figure S27.** The peak *R*, *EQE*, and *D*^*^ comparison for incident wavelengths of 450 nm, 637 nm, 785 nm, 1060 nm, and 1310 nm, showing a broadband visible to near-infrared photodetection ability.


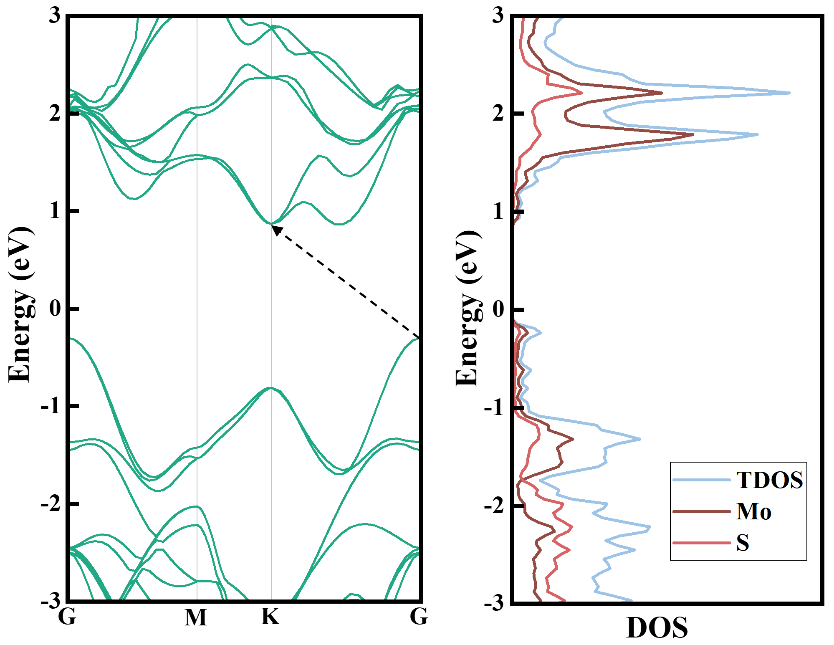


**Figure S28.** DFT calculation of the band structure and density of states (DOS) for 3L 3R-MoS_2_ without the introduction of sulfur vacancies.


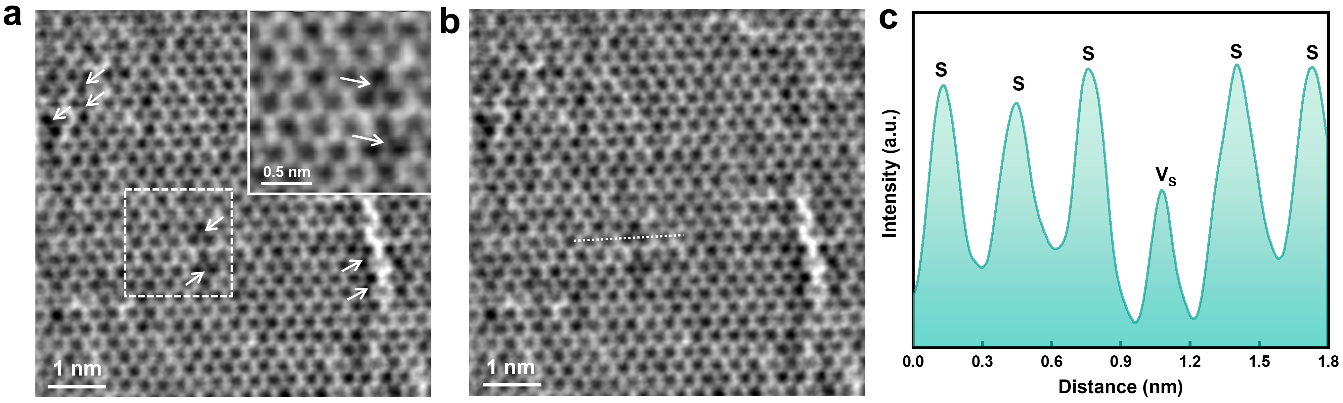


**Figure S29.** (a, b) STEM images of the 1L region of the 3L 3R-MoS_2_ nanoflake. The inset shows a magnified image for better identifying the position of the sulfur vacancies. The sulfur vacancies were indicated by the arrows in (a). (c) The line intensity profile acquired along the dashed line in (b), showing the darker spots ascribing to the sulfur vacancies.


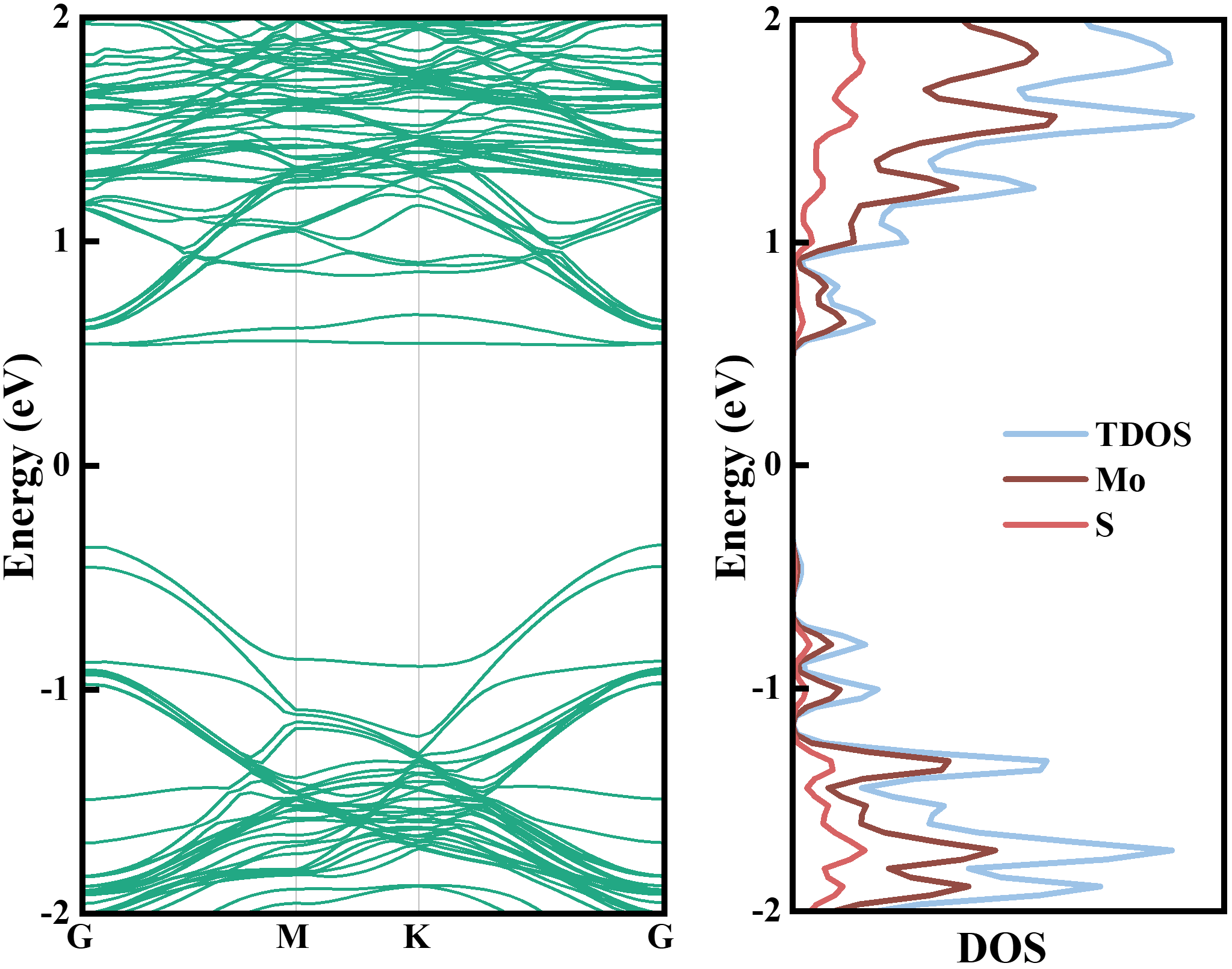


**Figure S30.** DFT calculation of the band structure and DOS for 3L 3R-MoS_2_ when sulfur vacancies (1 at%) were introduced.


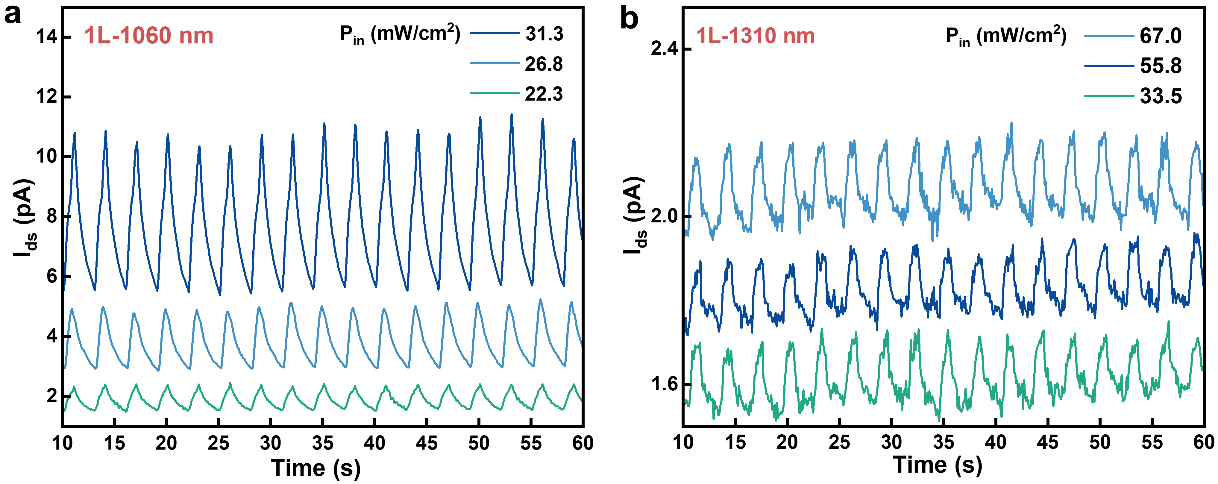


**Figure S31.** The temporal photoresponse of the 1L MoS_2_ device to (a) 1060 nm and (b) 1310 nm laser wavelengths with three distinct incident power densities.


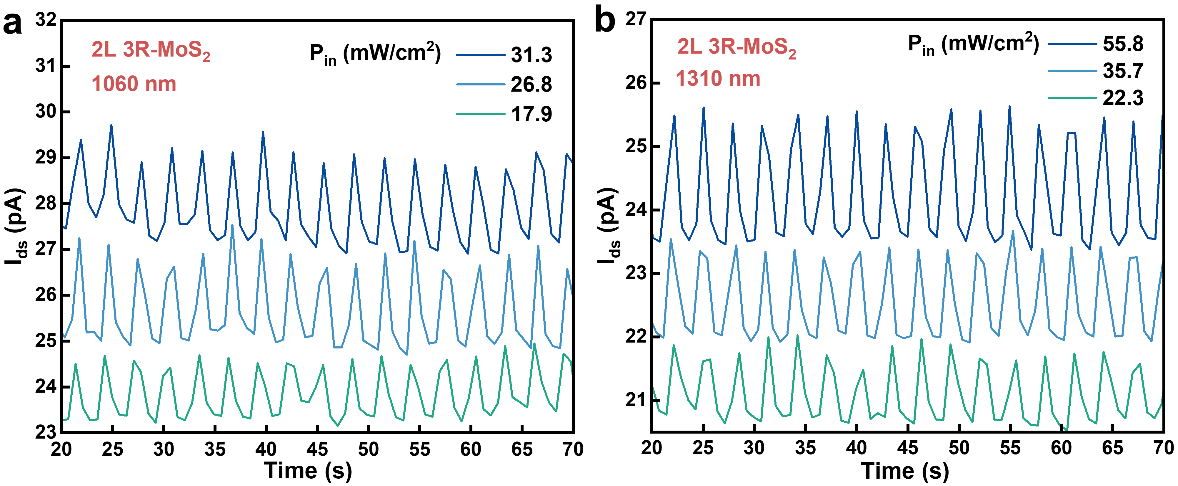


**Figure S32.** The temporal photoresponse of the 2L 3R-MoS_2_ device to (a) 1060 nm and (b) 1310 nm laser wavelengths with three distinct incident power densities.


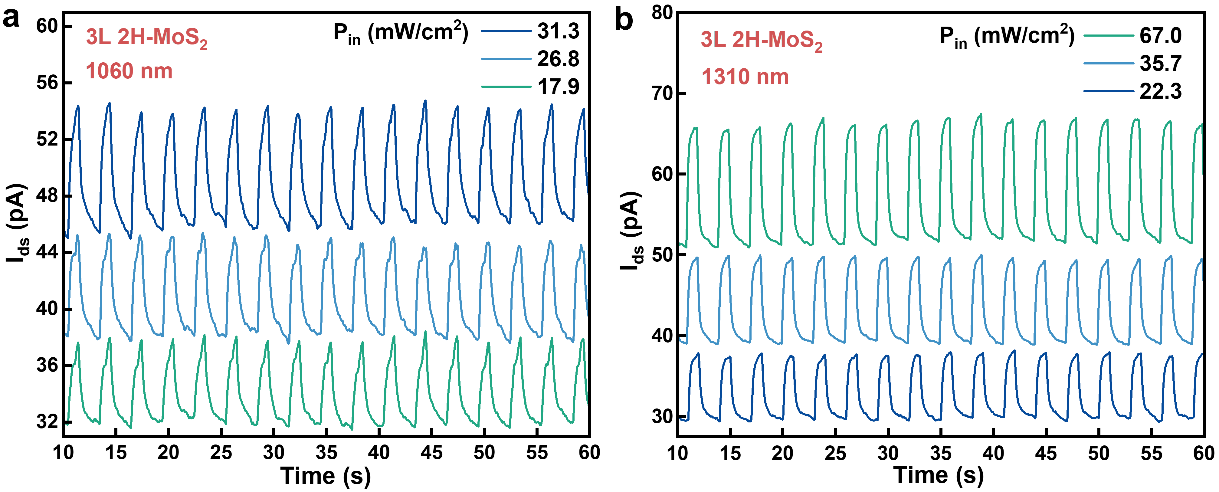


**Figure S33.** The temporal photoresponse of the 3L 2H-MoS_2_ device to (a) 1060 nm and (b) 1310 nm laser wavelengths with three distinct incident power densities.


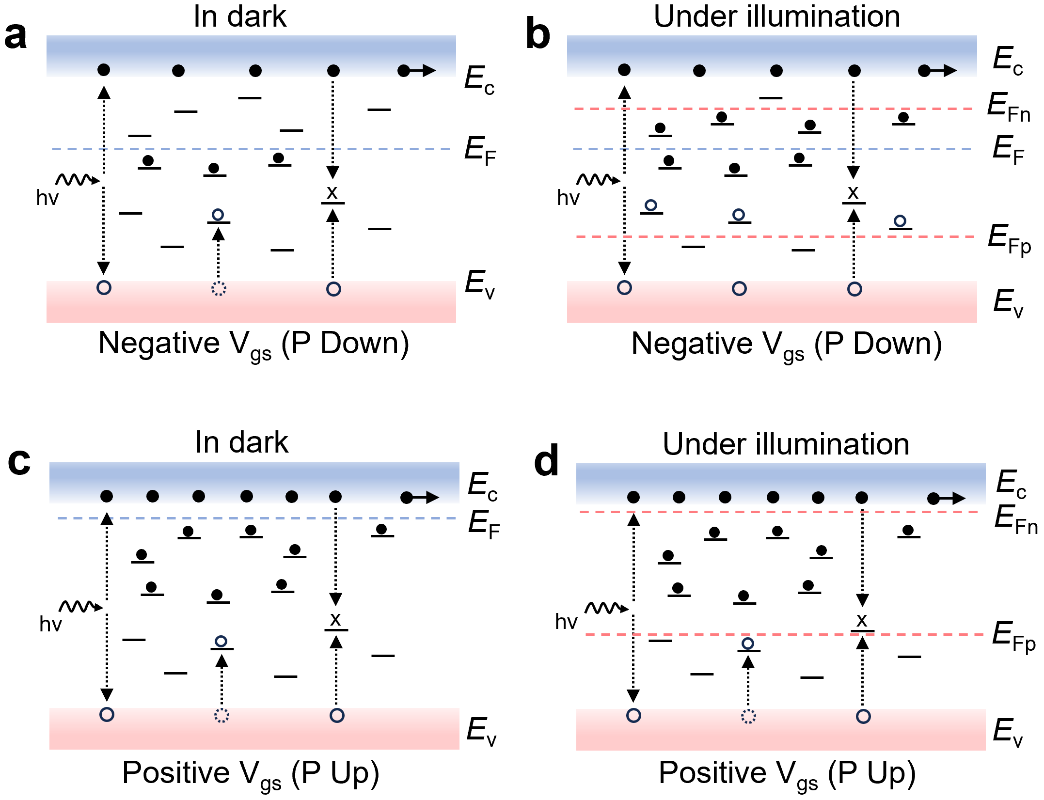


**Figure S34.** Gate-tunable photoresponse mechanisms. (a, b) At negative *V*_gs_, the free electrons are depleted leaving a large population of unoccupied trap states above the *E*_F_, which are filled by photogenerated carriers under illumination. (c, d) At positive *V*_gs_, *E*_F_ moves close to the *E*_c_ and the electron trap states are filled. The unoccupied hole trap states are gradually filled by the photogenerated carriers.


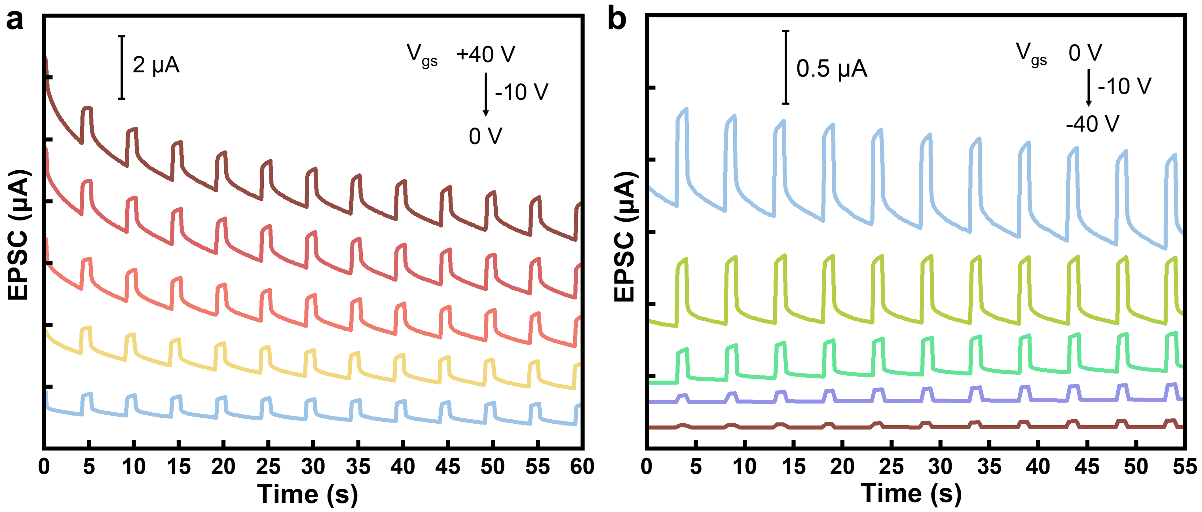


**Figure S35.** Temporal response of the device under 637 nm laser illumination and varied *V*_gs_ from -40 V to 40 V. (a) A declining trend of *I*_ds_ is observed for positive *V*_gs_ (40 to 0 V). (b) A rising trend in *I*_ds_ is observed for negative *V*_gs_ (-10 to -40 V ).


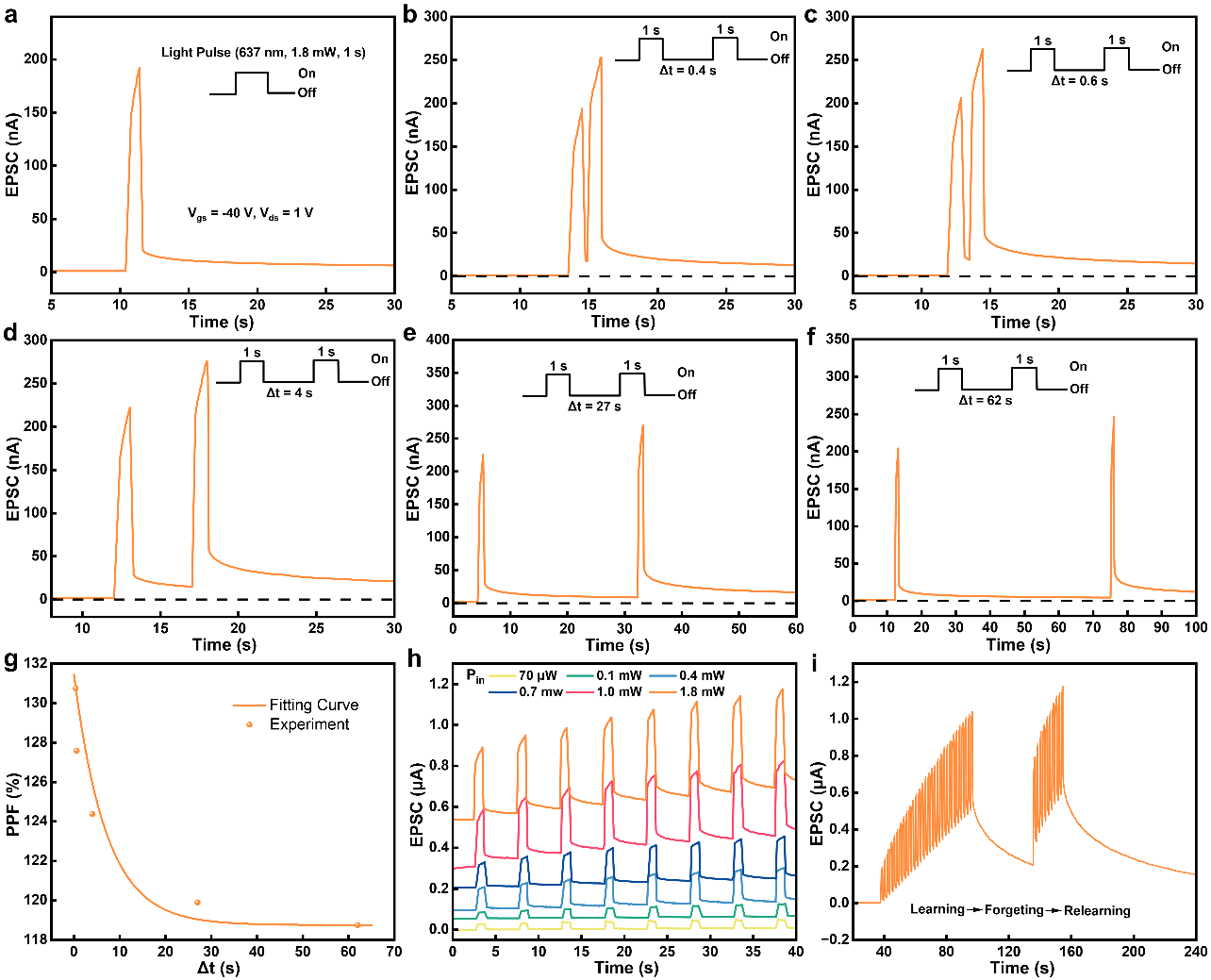


**Figure S36.** Neuromorphic optoelectronic synaptic behavior in 3L 3R-MoS_2_ device. (a) EPSC upon a single 637 nm light pulse was applied to the device. The device was set at *V*_gs_ = -40 V and *V*_ds_ = 1 V. (b-f) The PPF effect when a pair of 637 nm light pulses with an interval time of 0.4 s, 0.6 s, 4 s, 27 s, and 62 s, respectively, were applied to the device. (g) PPF ratio as a function of the pulse interval, defined as PPF = A_2_/A_1_, where A_2_ and A_1_ are the EPSC magnitudes after the second and first light pulse, respectively. (h) The plasticity transition from STP to LTP by increasing the laser power. (i) Learning, forgetting, and relearning processes emulated by the device, in which fewer light pulses are required to reach a higher EPSC level during the relearning process.


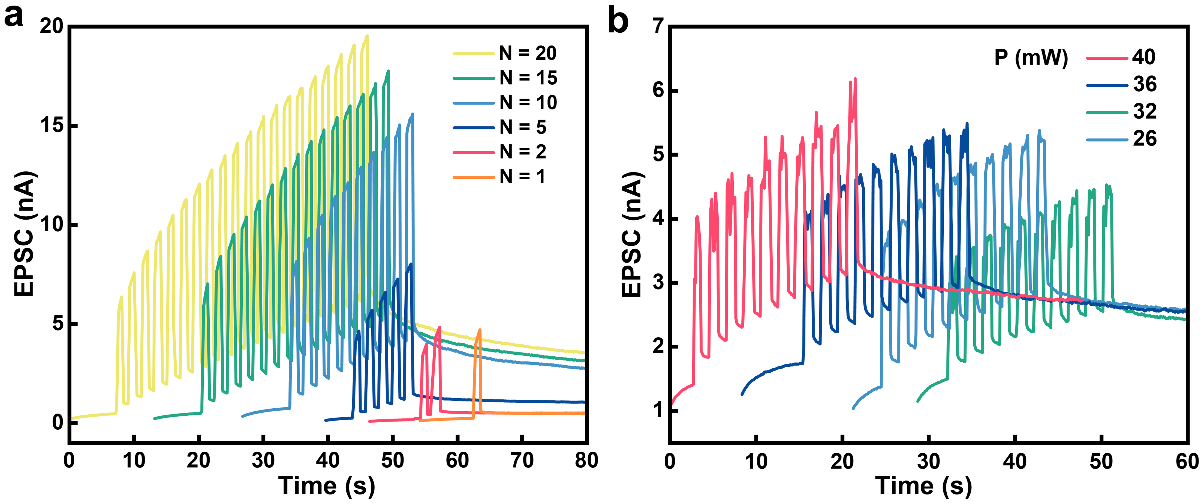


**Figure S37.** Neuromorphic optoelectronic synaptic behavior in 3L 3R-MoS_2_ device illuminated with a 785 nm laser. (a) The obviously increased EPSC by increasing the light pulse number (*N*). (b) The increased EPSC by increasing the laser power.


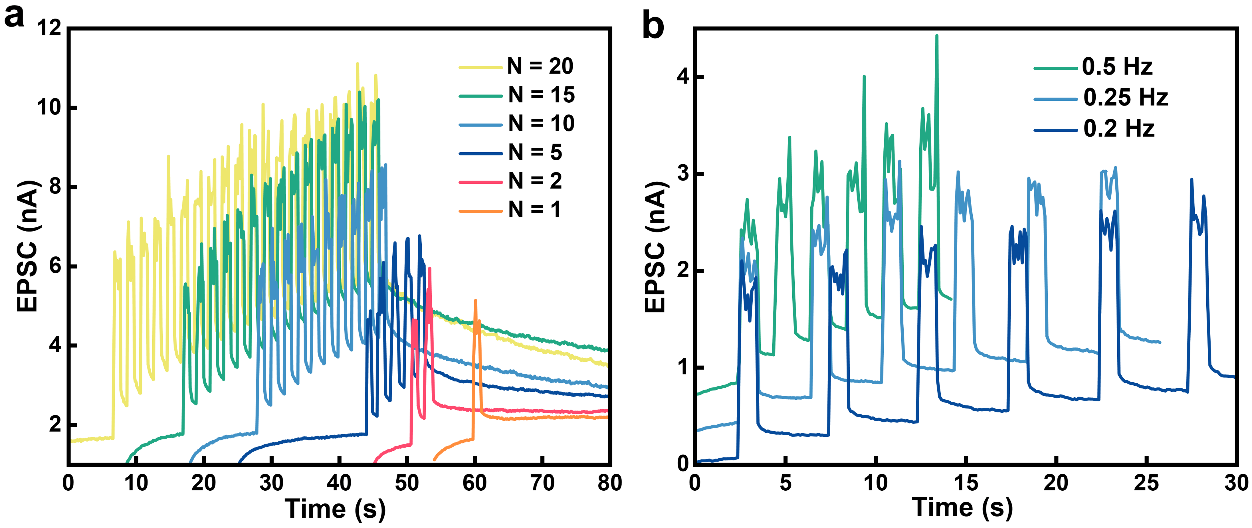


**Figure S38.** Neuromorphic optoelectronic synaptic behavior in 3L 3R-MoS_2_ device illuminated with a 1060 nm laser. (a) The obviously increased EPSC by increasing the light pulse number (*N*). (b) The increased EPSC by increasing the frequency of the light pulse.


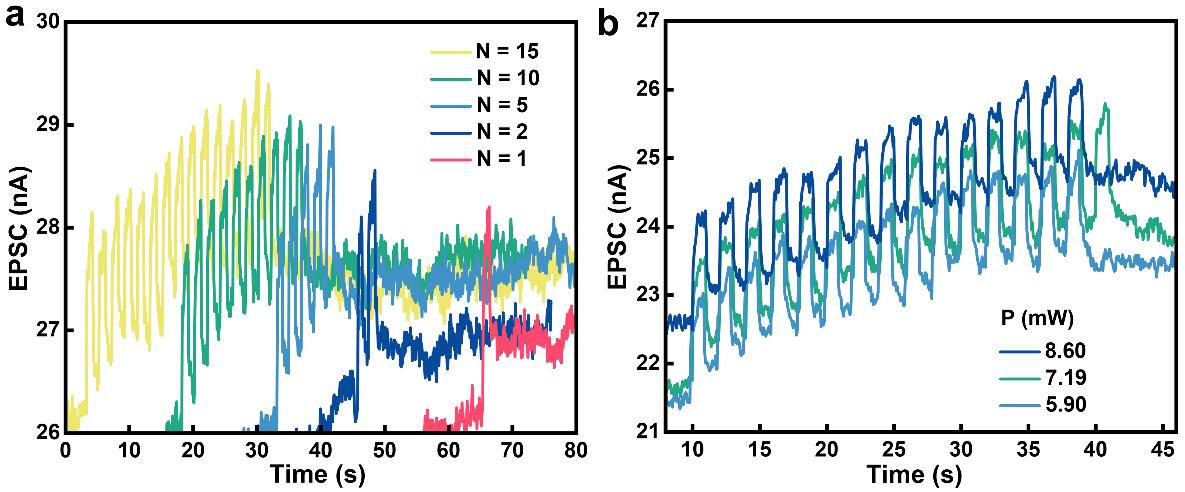


**Figure S39.** Neuromorphic optoelectronic synaptic behavior in 3L 3R-MoS_2_ device illuminated with a 1310 nm laser. (a) The obviously increased EPSC by increasing the light pulse number (*N*). (b) The increased EPSC by increasing the laser power.

**Table S1.** A comparison of the photodetector performance using 2D MoS_2_ as the photo-sensitive channel.

| Channel Materials | Wavelength (nm) | *V*_gs_ (V) | *V*_ds_ (V) | *R* (A/W) | *D** (Jones) | Ref. |
| --- | --- | --- | --- | --- | --- | --- |
| Few-layer MoS_2_ | 633 | -3 | 1 | 0.12 | 10^10^-10^11^ | [44] |
| 5L MoS_2_ film | 850 | 0 | 5 | 1.8 | 5×10^8^ | [45] |
| 1L MoS_2_ | 561 | -70 | 8 | 880 | 2.5×10^10^ | [46] |
| 1L MoS_2_ | 635 | 0 | 5 | 406 | 3.8×10^11^ | [47] |
| P(VDF-TrFE)/3L-MoS_2_ | 635 | 0 | 5 | 2570 | 2.2×10^12^ | [48] |
| 1L-2L MoS_2_ | 660 | - | - | 1×10^4^  (V_gs_ ~ 10 V) | 8×10^12^  (V_gs_ ~ -10 V) | [43] |
| 1L-8L MoS_2_ | 660 | - | - | 4×10^3^  (V_gs_ ~ -5 V) | 6×10^12^  (V_gs_ ~ -20 V) | [43] |
| 3L 3R-MoS_2_ | 450 | - | 1 | 1.8×10^6^  (V_gs_ ~ 40 V) | 1.13×10^15^  (V_gs_ ~ -10 V) | This work |
|  | 637 | - | 1 | 4.78×10^5^  (V_gs_ ~ 40 V) | 2.28×10^14^  (V_gs_ ~ -10 V) |  |

References

[1] H. Li, Q. Zhang, C. C. R. Yap, B. K. Tay, T. H. T. Edwin, A. Olivier, D. Baillargeat, *Adv. Funct. Mater.* **2012**, *22*, 1385.

[2] A. Splendiani, L. Sun, Y. Zhang, T. Li, J. Kim, C.-Y. Chim, G. Galli, F. Wang, *Nano Lett.* **2010**, *10*, 1271.

[3] D. J. Late, B. Liu, H. S. S. R. Matte, V. P. Dravid, C. N. R. Rao, *ACS Nano* **2012**, *6*, 5635.

[4] A. Di Bartolomeo, L. Genovese, F. Giubileo, L. Iemmo, G. Luongo, T. Foller, M. Schleberger, *2D Mater.* **2018**, *5*, 015014.

[5] X. Wang, P. Wang, J. Wang, W. Hu, X. Zhou, N. Guo, H. Huang, S. Sun, H. Shen, T. Lin, M. Tang, L. Liao, A. Jiang, J. Sun, X. Meng, X. Chen, W. Lu, J. Chu, *Adv. Mater.* **2015**, *27*, 6575.

[6] S. Zhang, Y. Liu, J. Zhou, M. Ma, A. Gao, B. Zheng, L. Li, X. Su, G. Han, J. Zhang, Y. Shi, X. Wang, Y. Hao, *Nanoscale Res. Lett.* **2020**, *15*, 157.

[7] A. Lipatov, P. Sharma, A. Gruverman, A. Sinitskii, *ACS Nano* **2015**, *9*, 8089.

[8] Z. Xian, C. Li, Y. Dong, M. Peng, Y. Yu, Y. Zhang, B. Huang, G. Zhong, S. Xie, J. Li, *Adv. Funct. Mater.* **2025**, *35*, 2409388.
